# Hepatitis E Virus Genotype 3 Diversity: Phylogenetic Analysis and Presence of Subtype 3b in Wild Boar in Europe

**DOI:** 10.3390/v7052704

**Published:** 2015-05-22

**Authors:** Ariel Vina-Rodriguez, Josephine Schlosser, Dietmar Becher, Volker Kaden, Martin H. Groschup, Martin Eiden

**Affiliations:** 1 Institute for Novel and Emerging Infectious Diseases, Friedrich-Loeffler-Institut, Südufer 10, 17493 Greifswald-Insel Riems, Germany; E-Mail: ArielVina.Rodriguez@fli.bund.de (A.V.-R.); josephine.schlosser@fli.bund.de (J.S.); martin.groschup@fli.bund.de (M.H.G.); 2 Micromun GmbH, Greifswald, Walther-Rathenau-Straße 49A, 17489 Greifswald, Germany; E-Mail: becher@micromun.de; 3 Institute of Infectology, Friedrich-Loeffler-Institut, Südufer 10, 17493 Greifswald-Insel Riems, Germany; E-Mail: kaden@gmx.de

**Keywords:** hepatitis E virus, HEV, genotype, subtype, phylogenetic analysis

## Abstract

An increasing number of indigenous cases of hepatitis E caused by genotype 3 viruses (HEV-3) have been diagnosed all around the word, particularly in industrialized countries. Hepatitis E is a zoonotic disease and accumulating evidence indicates that domestic pigs and wild boars are the main reservoirs of HEV-3. A detailed analysis of HEV-3 subtypes could help to determine the interplay of human activity, the role of animals as reservoirs and cross species transmission. Although complete genome sequences are most appropriate for HEV subtype determination, in most cases only partial genomic sequences are available. We therefore carried out a subtype classification analysis, which uses regions from all three open reading frames of the genome. Using this approach, more than 1000 published HEV-3 isolates were subtyped. Newly recovered HEV partial sequences from hunted German wild boars were also included in this study. These sequences were assigned to genotype 3 and clustered within subtype 3a, 3i and, unexpectedly, one of them within the subtype 3b, a first non-human report of this subtype in Europe.

## 1. Introduction

The Hepatitis E virus (HEV) is a causative agent of acute hepatitis in developing countries in Asia, Africa and Latin America where it is transmitted primarily via contaminated drinking water. Sporadic cases of HEV are reported in developed countries, partially imported by travelers from endemic areas, but there are also an increasing number of reports of autochthonous HIV infections. The transmission route of most of the autochthonous infections in industrialized countries still remains unclear. Reports of transfusion and transplant related infections exist [1,2,3], but accumulating evidence suggests that hepatitis E is a zoonotic disease with domestic pigs and wild boars being the main reservoirs. Moreover, the consumption of undercooked meat products poses a risk for HEV infection [4,5,6,7].

Studies on HEV RNA detection in animals revealed, that HEV is ubiquitous in domestic pigs and wild boars throughout Europe [8]. This includes the United Kingdom [9], France [10], Germany [11,12,13,14,15], Hungary [16], Italy [17,18], The Netherlands [19,20], Belgium [21], Spain [22], Slovenia [23], Czech Republic [24] and Sweden [25]. HEV infection in farmed pigs affects up to 80%–100% of the animals worldwide and usually occurs at the age of 2–4 months [26].

The virion is approximately 27–34 nm in diameter and most likely icosahedral. HEV has a positive sense single-stranded RNA genome of approximately 7.2 kb, which contains a short 5' untranslated region (UTR), a short 3' UTR and three open reading frames (ORF1, ORF2 and ORF3) [27]. The ORF1 encodes for viral non-structural proteins carrying domains with methyl transferase, helicase and replicase activities [28]. The ORF2 codes for the viral capsid protein of about 660 amino acids. The ORF3 is almost completely overlapped by the ORF2 and codes for a small phosphoprotein of about 114 amino acids, which is putatively responsible for the virion egress from infected cells [29].

A new proposed consensus for the HEV classification [30] divides the *Hepeviridae* family in two genera: *Orthohepevirus* and *Piscihepevirus*. The latter includes only isolates from cutthroat trout so far. The genus *Orthohepevirus* is further subdivided into four species: *Orthohepevirus* A with isolates from human, pig, wild boar, deer, mongoose, rabbit and camel, and *Orthohepevirus* B, C and D with avian and other mammal isolates. *Orthohepevirus* A is subsequently divided into at least six genotypes (HEV-1, HEV-2, *etc.*). HEV-1 and 2 include exclusively human HEV strains, whereas HEV-3 and 4 can also infect other animal species, particularly domestic pigs and wild boar. The separation in anthropotropic (HEV-1 and -2) and enzoonotic (HEV-3 and -4) forms may have occurred more than 500 year ago [31].

HEV-1 is found in Asia and Africa [32], whereas HEV-2 was first isolated in Mexico [33] and later in Africa [34]. HEV-4 includes strains from sporadic human HEV cases in Asia [35]. HEV-3 was isolated initially from human cases in the USA [36] and has been detected in all continents including Europe [37].

Since 2001, 2703 human HEV cases [38] have been reported in Germany, which include an increasing number of non-travel associated autochthonous cases. The origin of infection remains unclear for most of the autochthonous cases, however, often the suspected HEV sources are domestic pigs and wild boars [39]. Similar increases are also reported in other European countries [40].

The role of different HEV-3 genetic variants in the evolution of the disease [1,7,39,41,42], the possibility of tracking the routes of infection and the influences of human activity on it [43,44,45,46] are currently under study. The direct comparison of isolates is still hampered by the limited number of complete genome (CG) sequences available. Due to this limitation, the subtyping scheme proposed by Lu *et al.* [32] has been commonly used and have been supported by epidemiological and/or statistical analysis [4,43,45,47,48] but questions have arisen, partially due to the lack of commonly accepted reference sequences for some subtypes [1]. In response to this, and to the increasing number of partial sequences, the subtyping of genotype 3 strains was actualized in order to provide an update of the subtyping scheme of HEV-3 and of the set of reference sequences. We carried out the classification of newly recovered HEV isolates from German wild boar and detected HEV subtype 3b strains for the first time in animals in Europe and, possibly, the first from a wild animal outside Japan.

## 2. Materials and Methods

### 2.1. Samples and RNA Extraction

Blood samples were collected from wild boar hunted in Mecklenburg-Western Pomerania during the seasons 1996/1997 (955 samples) and 2005/2006 (58 samples). Liver samples were collected in 2009/2010 from 134 animals hunted in the region of Greifswald and from another five from Western Pomerania. All samples were stored at −80 °C prior to their use. RNA was extracted with the RNeasy Mini Kit (QIAGEN, Hilden, Germany) according to the manufacturer’s protocol. A synthetic RNA (IC) was used as internal extraction control [49].

### 2.2. Primers and Probe Design

For primer and probe design, an alignment of 351 HEV sequences was constructed using the Vector NTI Advanced v.10 (Invitrogen, Carlsbad, CA, USA), BioEdit v.7.0.5.3 [50] and MEGA v6 [51] software. This alignment was manually curated using both the nucleotide and the deduced amino acid sequences. Very similar sequences were not included (more than 99% identity). HEV-1, -2, -3 and -4 genotypes were included (with preference to genotype 3), covering all subtypes, and including 131 CG (48 of them cited by Lu *et al.* [32]) and 65 German HEV sequences (the accession numbers are included in Supplementary Table S01). For genotyping and subtyping, four sets of nested degenerated primers were selected from this alignment, which target different regions of the genome. Previously published primers [14] were used to amplify an RNA-dependent RNA polymerase (RdRp) region. A novel diagnostic quantitative real-time RT-PCR assay (qRT-PCR) that targets ORF3 was also designed, which we already used in a recently published work [52]. Primer and probes used are listed in Table 1 (nucleotide positions refer to FJ705359, strain wbGER27, a German wild boar isolate [14]) and were included in the GenBank sequences entries.

### 2.3. PCR

The diagnostic/screening RT-qPCR was performed using the QuantiTec Probe RT-PCR kit (QIAGEN) in 25 µL reaction volume. In all reactions, the final concentration of each primer was 0.8 µM, and of the probe 0.1 µM if present. A volume of 5 µL of the RNA eluate was added. The reverse transcription (RT) was carried out at 50 °C for 30 min, followed by denaturation/activation at 95 °C for 15 min. DNA was amplified immediately with 45 cycles at 95 °C (10 s), 55 °C (25 s) and 72 °C (25 s).

**Table 1:** Primer and probes used in this study. All nucleotide positions refer to FJ705359 (strain wbGER27, a German wild boar isolate). Abbreviations: open reading frame (ORF), hyper variable region (HVR), RNA dependent RNA polymerase (RdRp).

Region Name and Internal Length (nt)	Primer Name	Position	Sequence	Step	Product Length (bp)
***ORF1 -5´*** This study	**HEV.ORF1_F1**	33–58	CCCAYCAGTTYATWAAGGCTCCTGGC	RT-PCR	493
	**HEV.ORF1_R1**	497–525	TGCARDGARTANARRGCNAYNCCNGTCTC		
	**HEV.ORF1_F2**	98–126	AAYTCYGCCYTGGCGAATGCTGTGGTGGT	nested PCR	302
	**HEV.ORF1_R2**	377–399	CCVCGRGTNGGRGCRGWRTACCA		
***HVR*** (for genotype 3) This study	**HEV.HVR_F1**	2069–2091	TTYTCYCCTGGGCAYMTYTGGGA	RT-PCR	401
	**HEV.HVR_R1**	2441–2469	TTAACCARCCARTCACARTCYGAYTCAAA		
	**HEV.HVR_F2a**	2135–2157	ACYTGGTCHACATCTGGYTTYTC	nested PCR	263 or 293
	**HEV.HVR_F2b**	2165–2184	TTYTCCCCYCCTGAGGCGGC		
	**HEV.HVR_R2**	2405–2427	TACACCTTRGCSCCRTCRGGRTA		
***RdRp*** 280 (4312–4591) (Johne *et al.* 2010)	**HEV-cs**	4181–4203	TCGCGCATCACMTTYTTCCARAA	RT-PCR	469
	**HEV-cas**	4628–4650	GCCATGTTCCAGACDGTRTTCCA		
	**HEV-csn**	4287–4311	GTGCTCTGTTTGGCCCNTGGTTYMG	nested PCR	330
	**HEV-casn**	4592–4617	CCAGGCTCACCRGARTGYTTCTTCCA		
***ORF3*** 225 (5205–5429) This study	**HEV.ORF3_F1**	5126–5145	MGGKTRGAATGAATAACATG	RT-PCR	326 or 362
	**HEV.ORF3_R2**	5430–5451	GGCGCTGGGAYTGGTCRCGCCA		
	**HEV.ORF3_R1**	5467–5487	CAGYTGGGGYAGRTCGACGRC		
	**HEV.ORF3_F2**	5182–5204	GGGCTGTTCTGTTKYTGYTCYTC	nested PCR	219 or 269
	**HEV.ORF3_R2**	5430–5451	GGCGCTGGGAYTGGTCRCGCCA		
	**HEV.ORF3_R2a**	5382–5401	CGAGGGCGAGCTCCAGCCCC		
***modified Diagnostic-qPCR*** - This study and (Schlosser *et al.* 2014)	**HEV.Fa**	5278–5294	GTGCCGGCGGTGGTTTC	RT-qPCR	81
	**HEV.Fb**	5278–5296	GTGCCGGCGGTGGTTTCTG		
	**HEV.R**	5340–5359	GCGAAGGGGTTGGTTGGATG		
	**HEV.P**	5300–5320	FAM-TGACMGGGTTGATTCTCAGCC-BHQ1		
***ORF2*** 187 (6277–6488) This study	**HEV.ORF2_F1**	6205–6223	CDGCNACYCGBTTYATGAA	RT-PCR	393
	**HEV.ORF2_R1a**	6573–6598	GTKAGRGARAGCCAWAGYACATCATT		
	**HEV.ORF2_R1b**	6573–6598	GTRAGNGADAGCCACARRACATCATT		
	**HEV.ORF2_F2**	6276–6301	GCBYTHACNYTRTTYAAYCTTGCTGA	nested PCR	241
	**HEV.ORF2_R2**	6489–6517	TGYTCRTGYTGRTTRTCRTARTCYTGDAT		

The determination of the HEV RNA concentration was carried out using a standard curve according to a synthetic external calibrator. This calibrator encompassed the 81 bp sequence of the diagnostic qRT-PCR amplicon and included the T7 promoter sequence at the 5'-end for *in vitro* transcription. The RNA synthesis and DNA degradation were carried out by the Riboprobe^®^ Combination System—T3/T7 RNA Polymerase (Promega Corporation’s, Madison, WI, USA); and the QIAamp Viral RNA Mini Kit (QIAGEN) kit was used for RNA isolation (without carrier RNA). The RNA concentration was estimated with the Quant-It™ RNA Assay Kit, Broad Range (Invitrogen) and confirmed by endpoint dilution PCR.

For genotyping, the initial RT-qPCR was performed with the QuantiTec SYBR Green RT-PCR kit (QIAGEN) in 25 µL reaction volume using 5 µL of the sample RNA. The thermal profile applied was: 30 min at 50 °C for RT, 15 min 95 °C denaturation/activation followed by 45 cycles of 95 °C for 10 s, 55 °C for 25 s, 72 °C for 25 s and 80 °C for 5 s (with fluorescence reading). A final dissociation curve generation step was also included. Two microliters of the resulting solution was added to 23 µL of the Maxima™ SYBR Green/ROX qPCR Master Mix kit (Fermentas, Canada) containing the primers and the PCR was carried out under similar conditions (45 cycles of: 95 °C for 10 s, 55 °C for 25 s, 72 °C for 25 s and 80 °C for 5 s with a final dissociation curve generation step).

### 2.4. Sequencing, Phylogenetic Analysis and Classification

RT-PCR or nested-PCR products were directly sequenced with the corresponding forward and reverse PCR primers using the BigDye Terminator v1.1 Cycle Sequencing Kit on the DNA sequencer “3130 Genetic Analyzer” (Applied Biosystems, Waltham, MA, USA).

The newly generated sequences were manually inserted in the multi-alignment previously used for primer design. This multi-alignment was updated with new HEV sequences (NCBI, 2014-12-15), up to more than 1400 sequences (Supplementary Table S01), mainly genotype 3 (near 1300 sequences). This included all HEV-3 sequences longer 1000 nt, all HEV NCBI nucleotide entries with the keyword “Germany”, the 135 sequences cited by Lu *et al.* [32] and other sequences from around the word, but particularly from other European countries. The evolutionary history was inferred by using the Maximum Likelihood method based on the Kimura 2-parameter model [53]. The trees with the highest log are shown. The percentage of trees (boostrap values for 500 replicates for the first CG tree—and 100 replicates for all others trees) in which the associated taxa clustered together is shown next to the branches. Initial tree(s) for the heuristic search were obtained by applying the Neighbor-Joining (NJ) method to a matrix of pairwise distances estimated using the Maximum Composite Likelihood (MCL) approach. A discrete Gamma distribution was used to model evolutionary rate differences among sites (5 categories). The trees were drawn to scale, with branch lengths measured in the number of substitutions per site. Codon positions included were 1^st^ + 2^nd^ + 3^rd^. Evolutionary analyses were conducted in MEGA6 [51]. The alignment in FASTA format and the auxiliary worksheet for classification, selection and automatic labeling of sequences in MEGA are provided in Supplementary Files S01, S02 (updated versions are planned to be available from the authors). The sheet enables a quick selection of all the sequences spanning a given genomic region, which can be set in alignment coordinates (nt position) or by referencing to the sequences M73218 (Burma) or FJ705359 (wbGER27). Starting with the CG, and followed by the longest sequences, we built phylogenetic trees, and labeled each sequence as a “reference” to be used in subsequence classifications only if the tree reproduced the same topology as the tree for CG and if the clade was supported by bootstrap values of more than 70%. That is: HEV-3 sequences were labeled as “reference” only if the subtype was unambiguously determined. We assumed that all sequences from one strain represent the same genome sequence, and if one of them was labeled “reference”, all the others were also labeled.

## 3. Results

### 3.1. HEV RNA Detection

HEV RNA was detected in 32 out of 955 blood samples from 1996/97 and three out of 58 blood samples from 2005/2006, which suggests a virus prevalence of about 3.4% and 5.2%, respectively. In addition, HEV RNA was found in 14 out of 134 wild boar derived liver samples from the Greifswald region, giving a prevalence rate of about 10.4%. Finally, two wild boar liver samples (WS03-09 and WS05-09) from individual hunts were also positive. All HEV RNA positive samples were re-tested with the PCR for genotyping, and partial sequences from 12 animals could be recovered and subjected to phylogenetic analysis.

### 3.2. Phylogenetic Analyses

**Figure 1:**
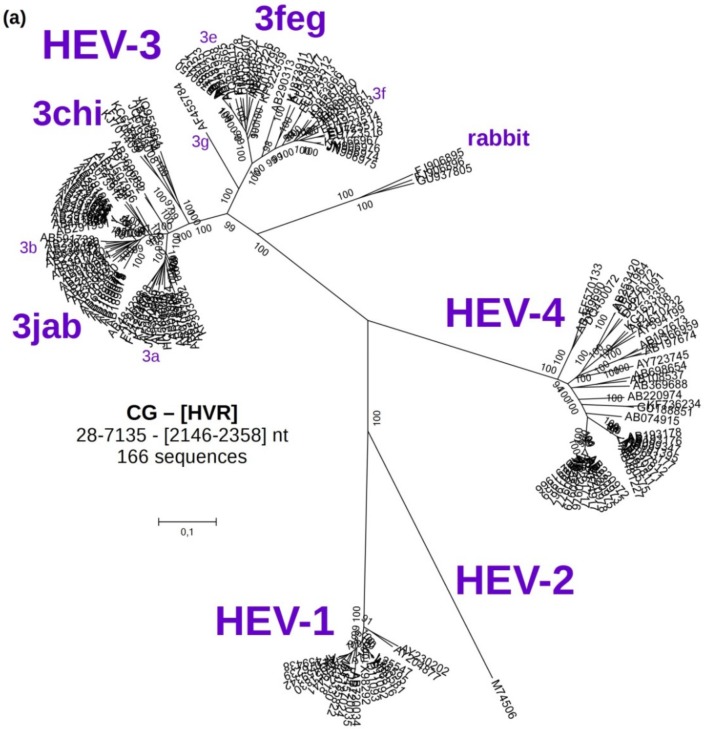

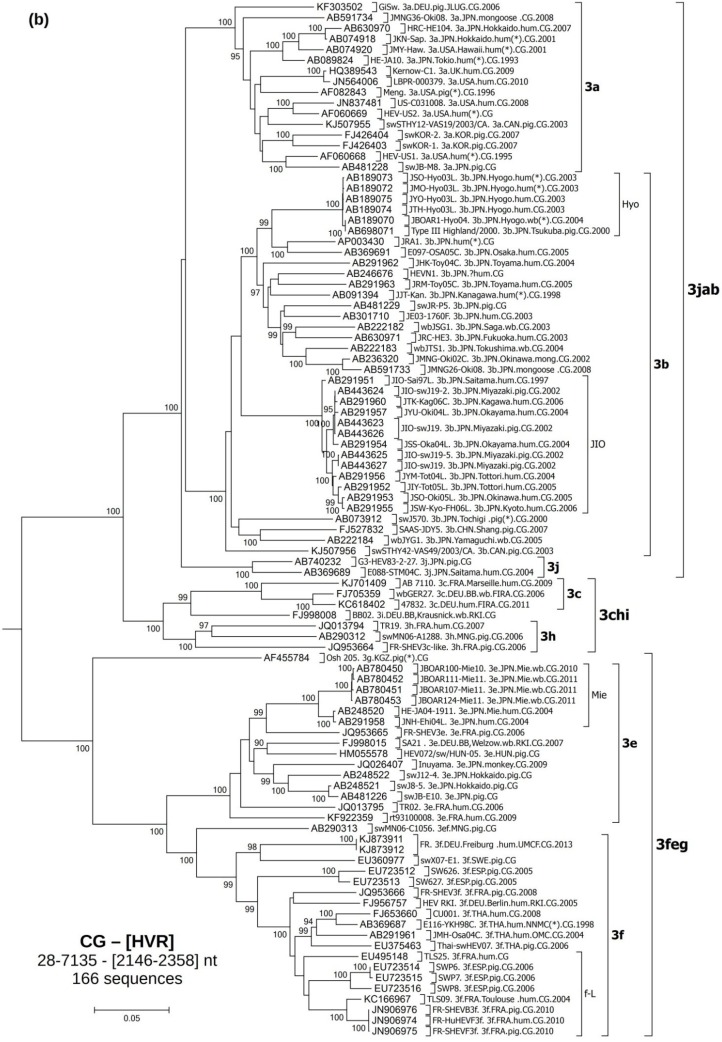
Molecular Phylogenetic analysis of 166 complete HEV genomes by Maximum Likelihood method based on the Kimura 2-parameter model. The percentage of trees (from 500 bootstrap replicates) in which the associated taxa clustered together is shown next to the branches when over 70%. Initial tree(s) for the heuristic search were obtained by applying the Neighbor-Joining method to a matrix of pairwise distances estimated using the Maximum Composite Likelihood (MCL) approach. A discrete Gamma distribution was used to model evolutionary rate differences among sites (five categories (+G, parameter = 0.5255)). The tree is drawn to scale, with branch lengths measured in the number of substitutions per site. All positions with less than 95% site coverage were eliminated. There were a total of 6868 positions in the final dataset. Evolutionary analyses were conducted in MEGA6. (**a**) Global view of the unrooted tree, which corresponds to Figure 4 in Lu *et al.* [32]; (**b**) Detailed view of the HEV-3 clade. (*)—references sequences cited by Lu; HRC-HE104—strain used in the HEV RNA WHO standard; wb—wild boar. See Supplementary Table 1 for more information.

A reference phylogenetic tree was constructed based on 166 CG sequences, including eight German HEV isolates and 98 strains of genotype 3 (Figure 1a, which corresponds to Figure 4 in [32]). The hypervariable region (HVR) (2146–2358 nt) was excluded from this analysis. All nucleotide positions refer to sequence M73218. This tree confirmed a good separation of the HEV-3 from all other HEV genotypes. The sequences clustered into four monophyletic groups: “3jab”, “3chi”, “3feg” and “rabbit”. A detailed overview of the HEV-3 clade is shown in Figure 1b.

The analysis was continued only with HEV-3, excluding the rabbit sequences, which form a well-separated clade.

**Figure 2:**
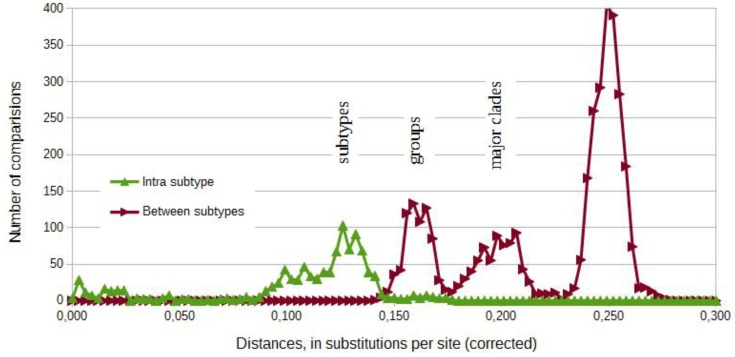
Frequency diagram of corrected distances between sequences from Figure 3a. It shows a possible separation of subtypes and suggests a possible definition of groups (3chi, 3jab and 3feg) and of major clades 3-I and 3-II (Norder, 2009). Pairwise distances were estimated using the Maximum Composite Likelihood (MCL) approach and grouped in intervals of 0.003. A discrete Gamma distribution was used to model evolutionary rate differences among sites (five categories). All positions with less than 95% site coverage were eliminated.

Comparisons amongst complete HEV-3 genomes display different levels of diversity within this genotype (Figure 2). The analysis of the frequency of corrected distances between sequences shows a possible separation around 0.14 substitutions per site. Sequences with lower differences belong to the same subtype. The graphic of the frequencies depicts two additional intermediary peaks, which are the basis for the definition of groups (3jab, 3chi and 3feg) and of major clades 3-I (3jab and 3chi) and 3-II (3feg) [54] (Figure 2).

**Figure 3:**
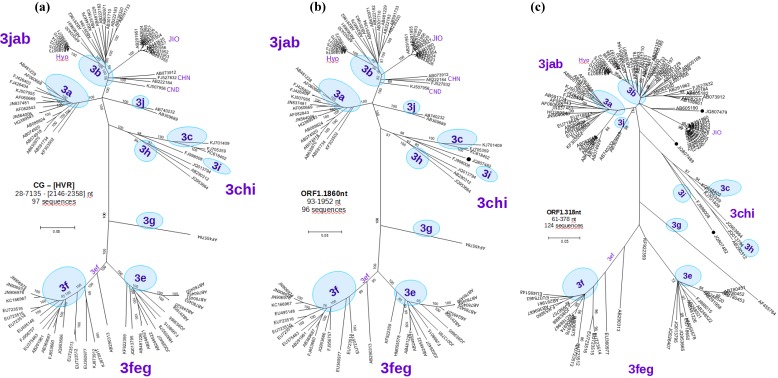
Molecular phylogenetic analysis of: (**a**) **CG-[HVR]**: 97 complete HEV-3 genomes, excluding the HVR (M73218:2146-2358 nt); (**b**) **ORF1.1860nt:** 96 partial HEV-3 genome sequences spanning the region 93–1952 nt, which correspond to a partial sequence JQ807482(●) obtained in this study from the animal WS05-09; (**c**) **ORF1.318nt**: 124 partial HEV-3 genome sequences spanning the region Burma.M73218: 61–378 nt approximately correspond to Figure 2 in [32]. Three of the sequences obtained in this study are included here (●): JQ807489, JQ807482 and JQ807479 (Bugewitz strain). Detailed view of the sequences used in b and c can be seen in supplementary Figures S01 and S02.

The newly obtained tree (Figure 3a) for complete HEV-3 genomes further segregated the sequences in subtypes clades 3j, 3a, 3b, 3c, 3h, 3e and 3f. This classification was supported by bootstrap values of 99%–100%. Subtypes 3i and 3g were represented by individual isolates. Ninety-six of these sequences were marked as “reference”. Two strains were marked 3ef. German strains were grouped in the 3a, 3c, 3i, 3e and 3f subtypes. This Figure represents our best approximation to the true topology and displays the reference structure for all subsequent trees.

**Figure 4:**
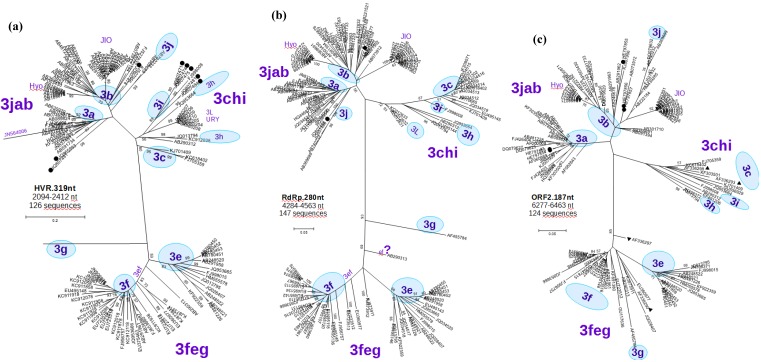
Molecular phylogenetic analysis of: (**a**) **HVR.319nt:** 126 partial HEV-3 genome sequences spanning the region 2094–2412 nt. The branches for sequences AF455784-3g and JN564006-3a are very large and have been truncated, including eight of the sequences obtained in this study (●); (**b**) **RdRp.280nt:** 147 partial HEV-3 genome sequences spanning the region 4284–4563 nt, including three of the sequences obtained in this study (●); (**c**) **ORF2.187nt:** 124 partial HEV-3 genome sequences spanning the region 6277–6463 nt, including five of the sequences obtained in this study (●), and stool pools from The Netherlands: 3c (▲) NLSW36 and NLSW105 and 3c+3f: (▼)NLSW20 and NLSW99 (see discussion). Detailed view of the sequences can be seen in supplementary Figures S07–S09.

**Figure 5:**
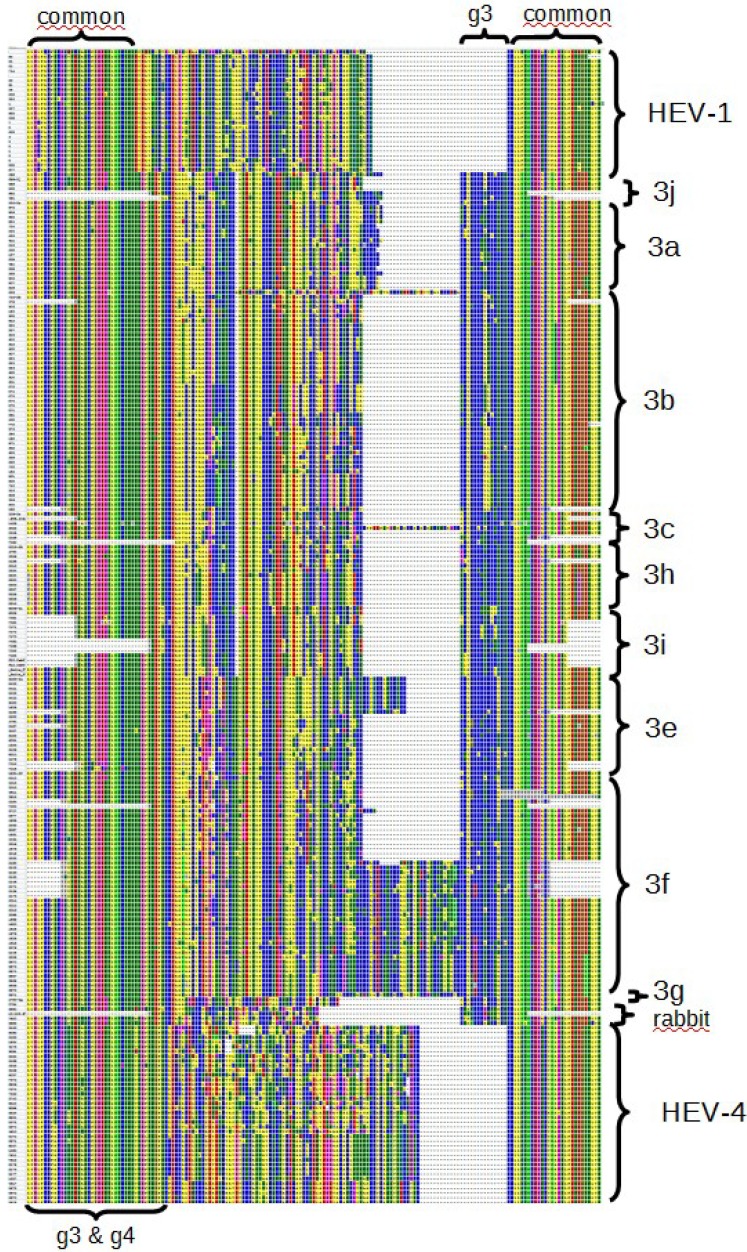
Scheme of the alignment of the deduced amino acid sequences including the HVR, which is flanked by conserved regions (some sequences only partially span this region and gaps in the conserved flanks are not part of the alignment). Obtained from the Alignment Explorer of MEGA6 (screenshot).

Each sequence in the alignment was analyzed by constructing a tree based on its exact (or near equal) length and including all the other sequences that span the region. More than 50 trees were analyzed, and as a result 68 more sequences were selected as “reference”, 48 of them longer than 1000 nt. Trees constructed for sequences more than 1500 nt length retained the same topology and support, with bootstrap values of at least 80% and each of the subtypes with more than one sequence. A representative tree based on 96 partial sequences spanning a region of 1860 nucleotide from position 93 to 1925 in the ORF1 (ORF1.1860nt) is depicted in Figure 3b.

Reduced sequence length maintains the same basic tree topology, but leads to a reduced or no bootstrap value support. This goes along with an increasing number of available sequences, better reflecting the HEV-3 diversity (Supplementary Figure S03). The tree based on a 318-nucleotide region from the 5’ section of the genome (position 61–378, ORF1.318nt), which includes the alignment of 124 partial sequences, is shown in Figure 3b. Newly recovered sequences from German wild boar isolates could be classified as subtype 3b (isolate WS03-09) and as subtype 3i (isolate WS05-09). Isolate WS34-10 was tentatively assigned to subtype 3b.

An overview of all the newly classified isolates from Mecklenburg-Western Pomerania and the corresponding accession numbers are depicted in Supplementary Table S02. A phylogenetic tree based on a 242-nucleotide region (positions 125–366 nt, ORF2.242nt) including 294 isolates is deposited at Supplementary Figure S03a.

The HVR (nucleotide position 2146–2358) was found to be particularly variable and was therefore manually aligned considering the deduced amino acid sequences. A scheme of a protein alignment of this region is shown in Figure 5. The longest stable HVR was found in strains of the subtype 3f, which were isolated from humans in France and Spanish pigs. In comparison, all other isolates contained shorter sequences without changing the coding frame. A tree based on a 319-nucleotide region (position 2094–2412) with 126 partial HEV-3 nucleotide sequences is shown in Figure 4a. This grouping assigned the strain WS03/09 to subtype 3b, the two isolates WS34-10 and WS35-10 were associated only weakly with subtype 3a and five isolates (WS05-09, WS21-10, 5160, 5304, 4322) clustered within subtype 3i.

A tree for 147 partial sequences within the RdRp region (280 nucleotide length, position 4284–4563, RdRp.280nt, Figure 4b, which approximately corresponds to Figure 6 presented by Lu *et al.* [32]) reproduced a similar topology compared to the CG. This tree confirmed the assignment of the strain WS03/09 to subtype 3b and a weak association of two isolates WS34-10 and WS28-10 to subtype 3a.

Finally, we carried out a phylogenetic analysis with sequences of the ORF3 (position 5180–5404, ORF3.225nt, Supplementary Figure S10/S11) and ORF2 (position 6277–6463, ORF2.187nt in Figure 4c, which partially correspond to Figure 5 in [32]) including 114 and 124 sequences, respectively. In both cases, monophyletic groups were not well supported leading to bootstrap values below 70% and in the case of the ORF3 without clear separation into subtypes. Thus, the subtypes were proposed (if possible) assuming the subtype of the nearest sequences. Using ORF3 derived sequences, we assigned three isolates to subtype 3a (isolate 8603, isolate 4701 and 4973), isolate WS03-09 to 3b, three isolates (WS28-10, WS34-10, WS35-10) weakly to 3b and two isolates to 3i (WS05-09, WS21-10) In the case of partial ORF2 derived sequences, two isolates segregated to subtype 3a (8603, 4701) and three isolates into subtype 3b (WS34-10, WS35-10, WS03-09).

In summary, twelve new isolates from Germany were analyzed using partial sequences from different regions of the genome (Supplementary Table S02). More than 1200 publicly available sequences, representing more than 1100 isolates, were subtyped accordingly and are listed in Supplementary Table S01. The classified strains are summarized by geographic region, subtype and host in Table 2.

**Table 2:** Subtype distribution of the 1109 isolates of genotype 3, represented by the 1283 sequences analyzed. Summarized by continent, country, and source—shown in order: human‘pig‘wild animal‘other. For example 1’2’3’4 means: 1 human isolate; 2 isolates from pig; 3 isolates from wild animals; and 4 from others sources (environment samples, water, *etc.*). As some isolates have undetermined subtypes and are not shown, not all rows or columns sum the real total. Due to various types of biases in the selection of the biological samples by the original authors and in our selection of sequences, the only approximate analysis possible was the comparison of the proportion of subtypes by host in different countries or regions. An alternative, graphical view is available at Supplementary Figure S13, which can also be explored interactively.

Origen	3	3jab				3chi				3feg					Total
		3a	3b	3d	3j	3h	3L	3i	3c	3e	3ef	3f	3g	3k	
**Africa**		****	****	****	****	**7**	****	****	****						**7**
CMR						0’4									0’4
CAN						0’3									0’3
**America**		**27**	**2**		**2**		**1**5								**47**
ARG					1		3’1								5’1
URY							10								10
BRA							0’1								0’1
CUB		7’6													7’6
CAN		2’1	1’1												3’2
MEX					0’1										0’1
USA		6’4’0’1													6’4’0’1
**Asia**	****	**18**	**67**	**3**	**3**	**3**	****	****	****	**13**	**1**	**5**	**1**	****	**114**
CHN			1’1	0’3											1’4
JPN		10’3’2	30’12’23		1’2					4’3’4					46’20’29
KOR		0’2													0’2
KGZ													0’1		0’1
MNG						0’1					0’1				0’2
NZL						1’1				1					2’1
THA												4’1			4’1
**Europe**	**8**	**59**	**2**	****	****	**10**	****	**25**	**277**	**326**	**2**	**208**	**9**	****	**940**
ESP		0’0’0’1				0’0’0’1				0’2		7’27’0’13			7’29’0’15
FRA			1			3’1			3’1	4’1’0’1		24’9			35’12’0’1
GRC										1		1			2’3
ITA						1			0’1			0’2			1’3
AUT		0’4						1’1							1’5
NLD		1’10							16’29’1	2’2’0’1		5’33’0’1			24’74’1’2
DEU	4	9’2’11	0’0’1			0’0’4		0’0’21	40’6’14	11’8’6	2	15’1’1	1		82’17’61
HUN		0’8’3								3’12’2		2			5’20’5
CZE										2		1’4	1’4’2		4’8’2
SRB		0’4											0’1’2		0’5
SVN														0’3	0’3
GBR	3	6							165	268		57			507
SWE	1							2	0’0’1			4’1			7’0’1’1
**Total**	**8**	**104**	**71**	**3**	**5**	**20**	**15**	**25**	**277**	**339**	**3**	**213**	**10**	**3**	**1109**

## 4. Discussion

The high sequence variability of HEV genomes is the central problem affecting the screening and diagnostic methods for the detection and quantification of the viral RNA. Therefore, the conserved ORF3 region offers a promising target for PCR assays (as reported by Jothikumar *et al.* [55]). Within this region, considering potential secondary structures, the high cg content, and trying to avoid the frequently observed non-specific signals with high Ct-values, we developed a new real-time RT-PCR assay (Table 1).

The HEV RNA detection rate of 3.4% in nearly 1000 sera collected from swine in 1996/97 and of 5.2% collected in 2005/2006 in the here presented study was similar to a previously reported 5.3% prevalence rate found in 189 samples collected in 1995/96 from the same region [12]. This indicates a constant circulation of HEV in this region. In addition, the prevalence of HEV in liver samples was 10.4%, which corresponds to the 14.9% [14] and 18.1% [13] found in wild boar derived liver samples from other regions in Germany.

The use of different and short genome regions for genotyping can lead to incongruences and provides insufficient evidence for establishing or refuting phylogenetic hypotheses [56]. Considering the given restriction, Lu *et al.* [32] proposed a comprehensive subtype scheme for the phylogenetic analysis of Hepatitis E virus, which has been commonly used. Nevertheless, Lu pointed to some incongruence in this scheme due to the use of different regions and to the small number of sequences within some subtypes that were available.

A major source of inconsistency during subtyping is the combined use of short sequences and the pooling of samples with a subsequent *in silico* concatenation of sequences. For example, Lu pointed out that the Arkell strain isolated from a pool of pig feces in Canada [57] is probably an artificial mixture of sequences, which could explain the inconsistent classification of this strain using different regions. Similarly, ORF1.242nt and ORF2.301nt sequences derived from pools of 20 to 60 pig faces [19] were used by Lu to define the 3c subtype (in the major clade I), but if only the ORF.148nt is analyzed, two of them distantly cluster 3f in the major clade II. Both 3c and 3f subtypes are common in Dutch pigs. Most of these inconsistences can be avoided using the original set (or a new consensus set) of reference sequences. Nevertheless, in general, this effect could appear without pooling of samples, due to co-infections and to true recombination between distant strains that are presumably rare events.

Another source of inconsistency is the lack, or insufficient number, of strains in some subtypes. The introduction of a new subtype based only on one single or a few short sequences can be error prone, due to laboratory artifacts, insufficient phylogenetic information, recombination, *etc.* For example, a new Hepatitis C virus (HCV) subtype (among other requisites) is created only when one complete genome (CG) and two other sufficiently informative sequences are available [58]. Basically, the CG will serve as a reference along the whole genome and the other two will determine the cluster, or prove the existence of a relatively recent common ancestor. This is an obvious problem within the HEV group 3chi: only 12 sequences longer than 1500 nt are available, from which only seven comprise CG (Supplementary Figure S12). Within the group 3chi, the best-described subtype appears to be subtype 3c (in the set of sequences we had already analyzed), with three CG and a large number of partial sequences. However, the 3i subtype is represented by only one CG, thus making it difficult to compare the sequences from different genomic regions that could be assigned to this subtype. Three CG were assigned to the subtype 3h, but they are highly divergent. It is important to note that the current poor structure of the group 3chi is not due to a rare detection of 3chi sequences, but rather to a relatively limited effort to obtain CG or nearly complete sequences. Most of the long HEV-3 sequences have been obtained in Japan (54 CG of 97 and 58 of 117 sequences longer 1500 nt), where 3chi apparently does not circulate.

Based on Lu’s classification scheme, we generated an updated phylogenetic tree with all newly available CG of genotype 3 and used the corresponding structure (Figure 3a) as template for subsequent classification of other strains based on partial sequences only. Our experimentally recovered partial sequences from different genomic regions, covering the 5’ ORF1, HVR, ORF3 and ORF2 (target regions selected in this study) and the RdRp regions [59] were originally selected to match that of the majority of the European sequences. The results show that partial sequences from our ORF1, HVR, RdRp and ORF2 regions generated trees with similar structures compared to the reference tree and can be used to subtype most sequences. In contrast, sequences from ORF3.225 are only partially suitable for classification up to the subtype level. In this context, the tree topology of Figure 3b and Figure 5 correspond to previously published trees (Figure 2 and Figure 6, Lu *et al.* [32])

The HVR is not a typical hypervariable region, but rather a genotypically diverse sequence [31]. The variability of this region has two components: (1) a higher mutation rate, and (2) insertions and/or deletions of one or two triplets or of much longer sequences (but maintaining the same reading frame) [60,61]. Taking this into account, it is almost trivial to find the right alignment manually and to decide whether the region should be included or not in the phylogenetic analyses. This alignment alone (Figure 5) allows an approximate reconstruction of the evolutionary history of the HEV genotypes and subtypes.

Based on the analysis of the 1652 nt part of the ORF2 not overlapping the ORF3 region, Purdy *et al.* [31] (including near 55 HEV-3 sequences) calculated the Time of the Most Recent Common Ancestor (TMRCA) of the four genotypes to be around the year 1475, and for HEV-3 and -4 around 1595. The TMRCA of major clades 3-I and 3-II (HEV-3 excluding the rabbit sequences) was determined near the year 1790, and for the clade 3-I (corresponding to 3chi and 3jab together) in 1865. Each group (3jab, 3chi and 3feg) has evolved roughly from 1900. While subtypes 3a and 3e have a TMRCA in 1945, the 3b and 3f+3ef are approximately 15 year older (without the 3ef, it is reduced for 3f to around 1960). Interestingly, the whole HEV-1 is only 100 years old. In a more recent analysis [43] using only the ORF2.301nt (including 208 HEV-3 sequences) the TMRCA for both major clades I and II together was dated to 1810 and for the clade I alone (3chi + 3jab groups) to 1895. Each group was correspondingly estimated: 3jab—1920; 3chi—1919; and 3feg—1889. For subtypes: 3a—1959; 3b—1944; 3f—1935; and 3e—1917 (include sequences not included by Purdy). The subtype 3d was the last separation, in 2002. Another study [45] shows compatible results and possible sources of minor discordance are discussed [43].

Not all possible methods of evolutionary tree reconstruction were thoroughly evaluated, but we noted that modeling evolutionary rate differences among sites have a major impact on the consistency of the results and that the tree generated with a ML method have longer internal branches and shorter terminal branches than with the NJ method, which is considered a good characteristic [62].

Partial sequences from twelve field isolates could be recovered and they all clustered within genotype 3. Three strains (4701, 4973, 8603) from the retrospective samples segregated to subtype 3a, which has been already detected in German autochthonous human infections [63], in wild boars around the city of Potsdam, in Brandenburg [14], as well as in human and pig samples from Bavaria [15]. Subtype 3a appears to be worldwide represented in samples from humans, pigs and wild animals (especially boar). In American samples, 3a could be the predominant and potential indigenous subtype (Supplementary Table S02), and import of USA pigs has been pointed to as a source of infection in South Korea and Japan [45].

Unexpectedly, the strain WS03-09 collected from an animal hunted in Western Pomerania clustered within subtype 3b for the four regions analyzed. In Europe, subtype 3b has not been detected in wild boars or domestic pig populations so far. This subtype probably originated from Japan [45] and has been mainly identified in humans, wild boars, domestic pigs and deer from that country. It has also been isolated from one Canadian pig and (reportedly) from humans and swine in Brazil [64]. In Europe, only one human isolate from France was grouped into subtype 3b [65]. We report here the first non-Japanese 3b isolate obtained from a wild animal.

WS 34/10, WS 35/10, and WS 28/10 could be classified 3jab, but could not be unambiguously subtyped and further investigations using longer sequences are needed to define if they cluster into existing subtypes (no -a or -b) or whether they define a new subtype within the 3jab group.

No sequences within subtype 3c were discovered in this study, although 3c appears to be specific for Central Europe and is the major subtype in Germany, Netherlands and recently United Kingdom, detected in humans, pigs and wild boars.

Sequences from animals WS 05/09, WS 21/10, 5160, 5304 and 4322 clustered within subtype 3i, which is closely related to the 3c, and could have similar distribution. Curiously, none of the 101 analyzed strains from The Netherlands was classified 3i, but 21 (all from wild board) out of 162 Germans strains were classified 3i. Until now, this subtype has been detected in Germany in only wild boars, but in Austria and Argentina has been also detected in humans.

Other subtypes were not detected, although especially subtype 3e and 3f are widely distributed in Europe (Table 2). The subtype 3e appears to be more widely distributed than 3c, including clusters of sequences from Japan and West Europe but it is more represented in Central Europe. In contrast, 3f sequences are more frequently found in Spain and France, and also found in other European countries. 3f has been also detected outside Europe in Thailand (two Japan patients were infected with this subtype after a trip to this country). Interesting, only one 3f strain was isolated from wild boar (from 94 total wild boar analyzed), but 204 were isolated from humans and domestic pigs (out of 998 from all subtypes). Finally, we recommend the use of partial sequences only when the obtained tree reproduces the same structure compared to the CG tree. Ideally, sequences with more than 1000 nt should be used for classification. In contrast, sequences below 200 nt should be avoided for subtyping. In particular, the commonly used ORF2.148nt, and the OFR2.171nt generate poorly structured trees. ORF3 sequences are sufficient for genotype, but not for subtype determination. HVR sequences should only be used for intra genotype comparisons, and alignments have to be checked manually, especially in the case of sequences with long insertions, which are impossible to be compared with the reference sequences. Do not define or modify subtypes based only on a single CG or only on short sequences (less than 1500 nt).

## 5. Conclusions

We designed RT-PCR assays for screening, quantification and genotyping of HEV-3 strains, and detected viral RNA in wild boar samples from Mecklenburg-Western Pomerania, Germany. Twelve strains clustered into subtypes 3a, 3i and, unexpectedly, also 3b, which is a common subtype in Japan, but has not been reported in animals in Europe. The phylogenetic trees based on our partial sequences of ORF1, RdRp, HVR and ORF2 regions reproduced similar topology as obtained from complete genome analysis and were useful for subtyping.

More than 30 different PCR fragments and the corresponding genomic regions have been used for genotyping and subtyping so far, which is a source of ambiguous subtyping schemes and inadequate classification. The presented study offers an updated set of reference sequences for the relatively simple and neutral subtype scheme proposed by Lu *et al.* [32], which could eliminates most of the existing incongruences and creates the basis for new hypotheses regarding the Hepatitis E epidemiology. A comprehensive subtyping of HEV-3 according to this classification scheme could enable a detailed view of the spread of HEV-3 strains among pigs, wild life and humans, and could allow determining the consequences of infections with different subtypes on humans and finally help limit the potential spread of the disease.

## Supplementary Information

### Tables

**Supplementary Table S01.** Global classification of Hepatitis E virus isolates using complete genomes (CG) or partial sequences from open reading frame 1 (ORF1), hyper variable region (HVR), RNA dependent RNA polymerase (RdRp), open reading frame 3 (ORF3) and open reading frame 2 (ORF2). All nucleotide positions refer to FJ705359 (wbGer27). Abbreviations: (*) reference sequences as cited by Lu *et al.* [32]; (**) strain used in the HEV RNA WHO standard (WHO, 2013); 3b.JIO—virulent strain from Japan; the sequences selected as “reference” are marked with an +; wb—wild boar; 3-letter country code after ISO 3166-1 alpha-3. When the information was available, this country code reflects the country from where the infection was imported. German isolates derived from Federal Institute for Risk Assessment (FIRA), Robert-Koch-Institut (RKI), Friedrich-Loeffler-Institut (FLI), Germany, University of Regensburg, Institute of Medical Microbiology and Hygiene (UReg), Bernhard Nocht Institute for Tropical Medicine (BNI) and University of Veterinary Medicine Hannover (TiHo). Burma (Myanmar, MMR) was conserved due to the widespread use in HEV related literature. mac-hum—isolated from macaques original inoculated witch human feces, or from both.

**Supplementary Table S02.** Subtype designation and accession numbers of sequences from isolates recovered in this study. *tentative assignments.

### Figures

**Supplementary Figure S01.** Detailed view of the sequences used in Figure 3b.

**Supplementary Figure S02.** Detailed view of the sequences used in Figure 3c.

**Supplementary Figure S03.** Molecular phylogenetic analysis of: (**a**) **ORF1.242nt (left):** 294 partial HEV-3 genome sequences spanning the region 125–366 nt, including all the sequences from Figure 2 in [32], a large number of European sequences, partial sequences from animals WS34-10: (●) JQ807489, and (●) JQ807482, and stool pools from Netherlands: 3c (▲) NLSW36 and NLSW105 and 3c + 3f: (▼)NLSW20 and NLSW99 (see discussion); (**b**) **ORF2.280nt (center):** 665 partial HEV-3 genome sequences spanning the region 6017–6296 nt including near 500 sequences from England and Wales (Ijaz *et al.* 2014), and from the same stool pools from The Netherlands: (▲) and (▼); (**c**) **ORF2.241nt (right):** 156 partial HEV-3 genome sequences spanning the region 5596–5836 nt. A detailed view of all the sequences used is given in Supplementary Figures S04, S05 and S06, respectively.

**Supplementary Figure S04.** Detailed view of the sequences used in Supplementary Figure S03a.

**Supplementary Figure S05.** Detailed view of the sequences used in Supplementary Figure S03b.

**Supplementary Figure S06.** Detailed view of the sequences used in Supplementary Figure S03c.

**Supplementary Figure S07.** Detailed view of the sequences used in Figure 4a.

**Supplementary Figure S08.** Detailed view of the sequences used in Figure 4b.

**Supplementary Figure S09.** Detailed view of the sequences used in Figure 4c.

**Supplementary Figure S10.** Molecular phylogenetic analysis of: (a) **ORF3.225nt:** 114 partial HEV-3 genome sequences spanning the region 5180–5404 nt, including sequences obtained in this study (●).

**Supplementary Figure S11.** Detailed view of the sequences used in in Supplementary Figure S10.

**Supplementary Figure S12.** Detailed view of the 3chi group from three partial genomic regions.

**Supplementary Figure S13.** Geographical distribution of subtypes by host (human, domestic pig, or wild animals). This information can be also interactively explored at: https://public.tableau.com/views/HEV-g3/Story3?:embed=y&:showTabs=y&:display_count=yes.

### Supplementary Files

**Supplementary File S01:** **HEV-A.fasta.zip.** Multialignment in FASTA format, compressed.

**Supplementary File S02:** **HEVsubtypingMEGAut.xlsx.** Auxiliary worksheet for classification, selection and automatic labeling of sequences in MEGA.
