# Supplementary material for: Hepatitis E Virus Genotype 3 Diversity: Phylogenetic Analysis and Presence of Subtype 3b in Wild Boar in Europe
Source: Viruses. 2015 May 22;7(5):2704–26. doi: 10.3390/v7052704 (PMC4452927; doi:10.3390/v7052704)
Supplement: Supplementary file 1 [file viruses-07-02704-s001.zip › viruses-84017-supplementary/Supplem.Fig S01. for Fig3b-center.ORF1.1860nt (Burma.M73218 - 93-1952 nt) details.pdf]

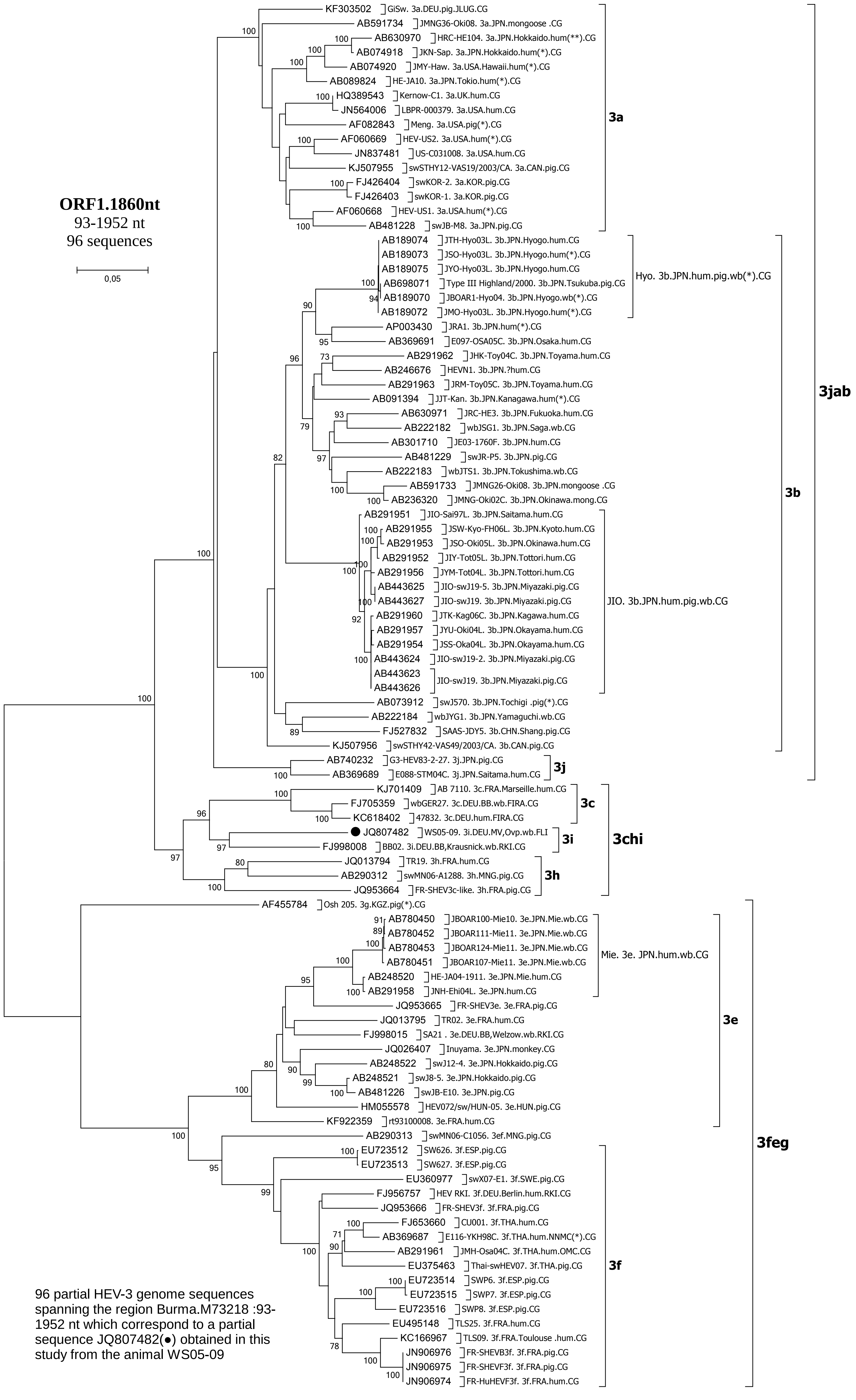

ORF1.1860nt
93-1952 nt
96 sequences
0,05
KF303502 GiSw. 3a.DEU.pig.JLUG.CG
AB591734 JMNG36-Oki08. 3a.JPN.mongoose .CG
AB630970 HRC-HE104. 3a.JPN.Hokkaido.hum(**).CG
AB074918 JKN-Sap. 3a.JPN.Hokkaido.hum(*).CG
AB074920 JMY-Haw. 3a.USA.Hawaii.hum(*).CG
AB089824 HE-JA10. 3a.JPN.Tokio.hum(*).CG
HQ389543 Kernow-C1. 3a.UK.hum.CG
JN564006 LBPR-000379. 3a.USA.hum.CG
AF082843 Meng. 3a.USA.pig(*).CG
AF060669 HEV-US2. 3a.USA.hum(*).CG
JN837481 US-C031008. 3a.USA.hum.CG
KJ507955 swSTHY12-VAS19/2003/CA. 3a.CAN.pig.CG
FJ426404 swKOR-2. 3a.KOR.pig.CG
FJ426403 swKOR-1. 3a.KOR.pig.CG
AF060668 HEV-US1. 3a.USA.hum(*).CG
AB481228 swJB-M8. 3a.JPN.pig.CG
3a
AB189074 JTH-Hyo03L. 3b.JPN.Hyogo.hum.CG
AB189073 JSO-Hyo03L. 3b.JPN.Hyogo.hum(*).CG
AB189075 JYO-Hyo03L. 3b.JPN.Hyogo.hum.CG
AB698071 Type III Highland/2000. 3b.JPN.Tsukuba.pig.CG
AB189070 JBOAR1-Hyo04. 3b.JPN.Hyogo.wb(*).CG
AB189072 JMO-Hyo03L. 3b.JPN.Hyogo.hum(*).CG
Hyo. 3b.JPN.hum.pig.wb(*).CG
AP003430 JRA1. 3b.JPN.hum(*).CG
AB369691 E097-OSA05C. 3b.JPN.Osaka.hum.CG
AB291962 JHK-Toy04C. 3b.JPN.Toyama.hum.CG
AB246676 HEVN1. 3b.JPN.?hum.CG
AB291963 JRM-Toy05C. 3b.JPN.Toyama.hum.CG
AB091394 JJT-Kan. 3b.JPN.Kanagawa.hum(*).CG
AB630971 JRC-HE3. 3b.JPN.Fukuoka.hum.CG
AB222182 wbJSG1. 3b.JPN.Saga.wb.CG
AB301710 JE03-1760F. 3b.JPN.hum.CG
AB481229 swJR-P5. 3b.JPN.pig.CG
AB222183 wbJTS1. 3b.JPN.Tokushima.wb.CG
AB591733 JMNG26-Oki08. 3b.JPN.mongoose .CG
AB236320 JMNG-Oki02C. 3b.JPN.Okinawa.mong.CG
AB291951 JIO-Sai97L. 3b.JPN.Saitama.hum.CG
AB291955 JSW-Kyo-FH06L. 3b.JPN.Kyoto.hum.CG
AB291953 JSO-Oki05L. 3b.JPN.Okinawa.hum.CG
AB291952 JIY-Tot05L. 3b.JPN.Tottori.hum.CG
AB291956 JYM-Tot04L. 3b.JPN.Tottori.hum.CG
AB443625 JIO-swJ19-5. 3b.JPN.Miyazaki.pig.CG
AB443627 JIO-swJ19. 3b.JPN.Miyazaki.pig.CG
AB291960 JTK-Kag06C. 3b.JPN.Kagawa.hum.CG
AB291957 JYU-Oki04L. 3b.JPN.Okayama.hum.CG
AB291954 JSS-Oka04L. 3b.JPN.Okayama.hum.CG
AB443624 JIO-swJ19-2. 3b.JPN.Miyazaki.pig.CG
AB443623
AB443626 JIO-swJ19. 3b.JPN.Miyazaki.pig.CG
JIO. 3b.JPN.hum.pig.wb.CG
AB073912 swJ570. 3b.JPN.Tochigi .pig(*).CG
AB222184 wbJYG1. 3b.JPN.Yamaguchi.wb.CG
FJ527832 SAAS-JDY5. 3b.CHN.Shang.pig.CG
KJ507956 swSTHY42-VAS49/2003/CA. 3b.CAN.pig.CG
3b
3jab
AB740232 G3-HEV83-2-27. 3j.JPN.pig.CG
AB369689 E088-STM04C. 3j.JPN.Saitama.hum.CG
3j
KJ701409 AB 7110. 3c.FRA.Marseille.hum.CG
FJ705359 wbGER27. 3c.DEU.BB.wb.FIRA.CG
KC618402 47832. 3c.DEU.hum.FIRA.CG
3c
● JQ807482 WS05-09. 3i.DEU.MV,Ovp.wb.FLI
FJ998008 BB02. 3i.DEU.BB,Krausnick.wb.RKI.CG
3i
JQ013794 TR19. 3h.FRA.hum.CG
AB290312 swMN06-A1288. 3h.MNG.pig.CG
JQ953664 FR-SHEV3c-like. 3h.FRA.pig.CG
3h
3chi
AF455784 Osh 205. 3g.KGZ.pig(*).CG
AB780450 JBOAR100-Mie10. 3e.JPN.Mie.wb.CG
AB780452 JBOAR111-Mie11. 3e.JPN.Mie.wb.CG
AB780453 JBOAR124-Mie11. 3e.JPN.Mie.wb.CG
AB780451 JBOAR107-Mie11. 3e.JPN.Mie.wb.CG
AB248520 HE-JA04-1911. 3e.JPN.Mie.hum.CG
AB291958 JNH-Ehi04L. 3e.JPN.hum.CG
Mie. 3e. JPN.hum.wb.CG
JQ953665 FR-SHEV3e. 3e.FRA.pig.CG
JQ013795 TR02. 3e.FRA.hum.CG
FJ998015 SA21 . 3e.DEU.BB,Welzow.wb.RKI.CG
JQ026407 Inuyama. 3e.JPN.monkey.CG
AB248522 swJ12-4. 3e.JPN.Hokkaido.pig.CG
AB248521 swJ8-5. 3e.JPN.Hokkaido.pig.CG
AB481226 swJB-E10. 3e.JPN.pig.CG
HM055578 HEV072/sw/HUN-05. 3e.HUN.pig.CG
KF922359 rt93100008. 3e.FRA.hum.CG
3e
AB290313 swMN06-C1056. 3ef.MNG.pig.CG
EU723512 SW626. 3f.ESP.pig.CG
EU723513 SW627. 3f.ESP.pig.CG
EU360977 swX07-E1. 3f.SWE.pig.CG
FJ956757 HEV RKI. 3f.DEU.Berlin.hum.RKI.CG
JQ953666 FR-SHEV3f. 3f.FRA.pig.CG
FJ653660 CU001. 3f.THA.hum.CG
AB369687 E116-YKH98C. 3f.THA.hum.NNMC(*).CG
AB291961 JMH-Osa04C. 3f.THA.hum.OMC.CG
EU375463 Thai-swHEV07. 3f.THA.pig.CG
EU723514 SWP6. 3f.ESP.pig.CG
EU723515 SWP7. 3f.ESP.pig.CG
EU723516 SWP8. 3f.ESP.pig.CG
EU495148 TLS25. 3f.FRA.hum.CG
KC166967 TLS09. 3f.FRA.Toulouse .hum.CG
JN906976 FR-SHEVB3f. 3f.FRA.pig.CG
JN906975 FR-SHEVF3f. 3f.FRA.pig.CG
JN906974 FR-HuHEVF3f. 3f.FRA.hum.CG
3f
3feg
96 partial HEV-3 genome sequences spanning the region Burma.M73218 :93-1952 nt which correspond to a partial sequence JQ807482(●) obtained in this study from the animal WS05-09
