# Supplementary material for: Hepatitis E Virus Genotype 3 Diversity: Phylogenetic Analysis and Presence of Subtype 3b in Wild Boar in Europe
Source: Viruses. 2015 May 22;7(5):2704–26. doi: 10.3390/v7052704 (PMC4452927; doi:10.3390/v7052704)
Supplement: Supplementary file 1 [file viruses-07-02704-s001.zip › viruses-84017-supplementary/Supplem.Fig S03. 3 trees.docx]

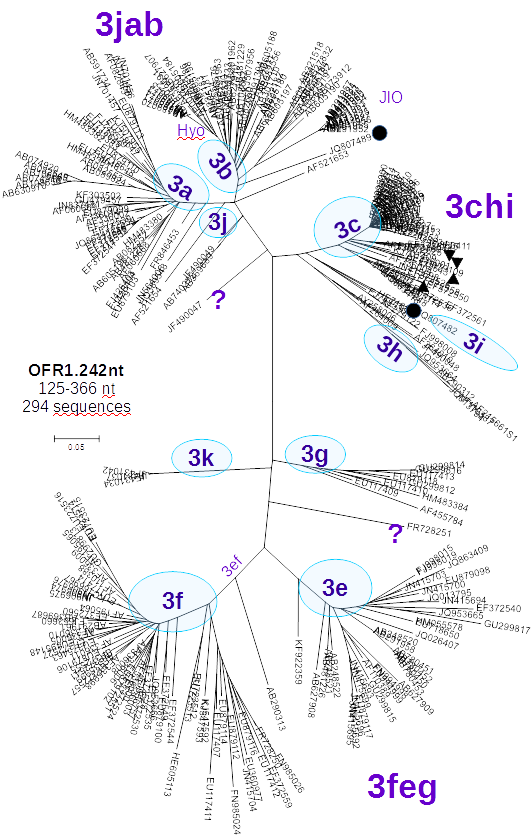

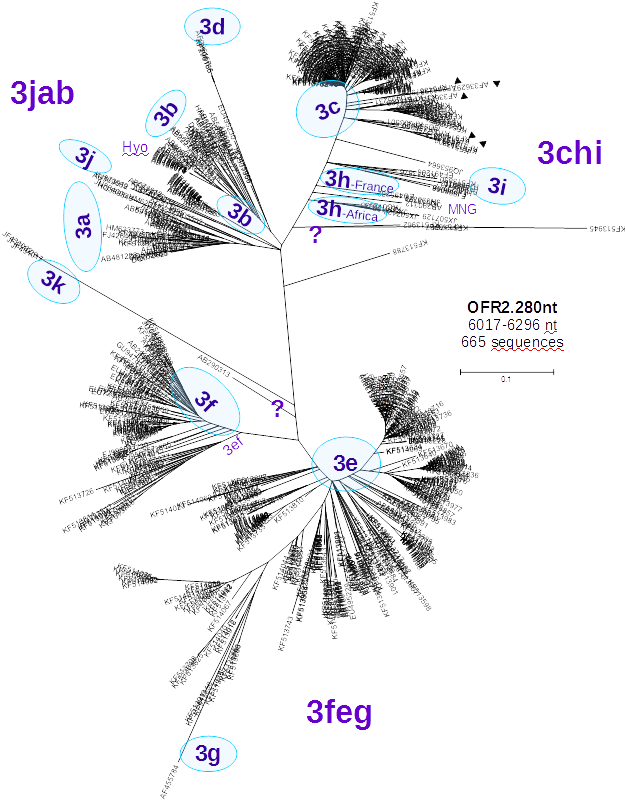

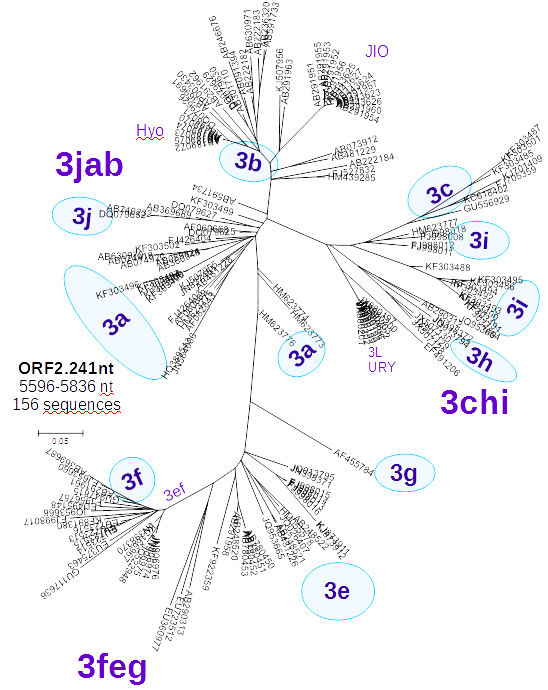


**(a)**

**(c)**

**(b)**

**Suplementary Figure S03.** Molecular phylogenetic analysis of: (a) **ORF1.242nt (left):** 294 partial HEV-3 genome sequences spanning the region 125-366 nt including all the sequences from figure 2 in [[32](#_ENREF_32)], a large number of European sequences, partial sequences from animals WS34-10: (●) JQ807489, and (●) JQ807482, and stool pools from Netherlands: 3c (▲) NLSW36 and NLSW105 and 3c+3f: (▼)NLSW20 and NLSW99 [see discussion]; (b) **ORF2.280nt (center):** 665 partial HEV-3 genome sequences spanning the region 6017-6296 nt including near 500 sequences from England and Wales (Ijaz et. al 2014), and from the same stool pools from Netherlands: (▲) and (▼); (c) **ORF2.241nt (right):** 156 partial HEV-3 genome sequences spanning the region 5596-5836 nt. Detailed view of all the sequences used can be seen in Supplementary Figures S04, S05 and S06 respectively.
