# Supplementary figures and images for: Hepatitis E Virus Genotype 3 Diversity: Phylogenetic Analysis and Presence of Subtype 3b in Wild Boar in Europe
Source: Viruses. 2015 May 22;7(5):2704–26. doi: 10.3390/v7052704 (PMC4452927; doi:10.3390/v7052704)

**ORF1.318nt**  
61-378 nt  
124 sequences

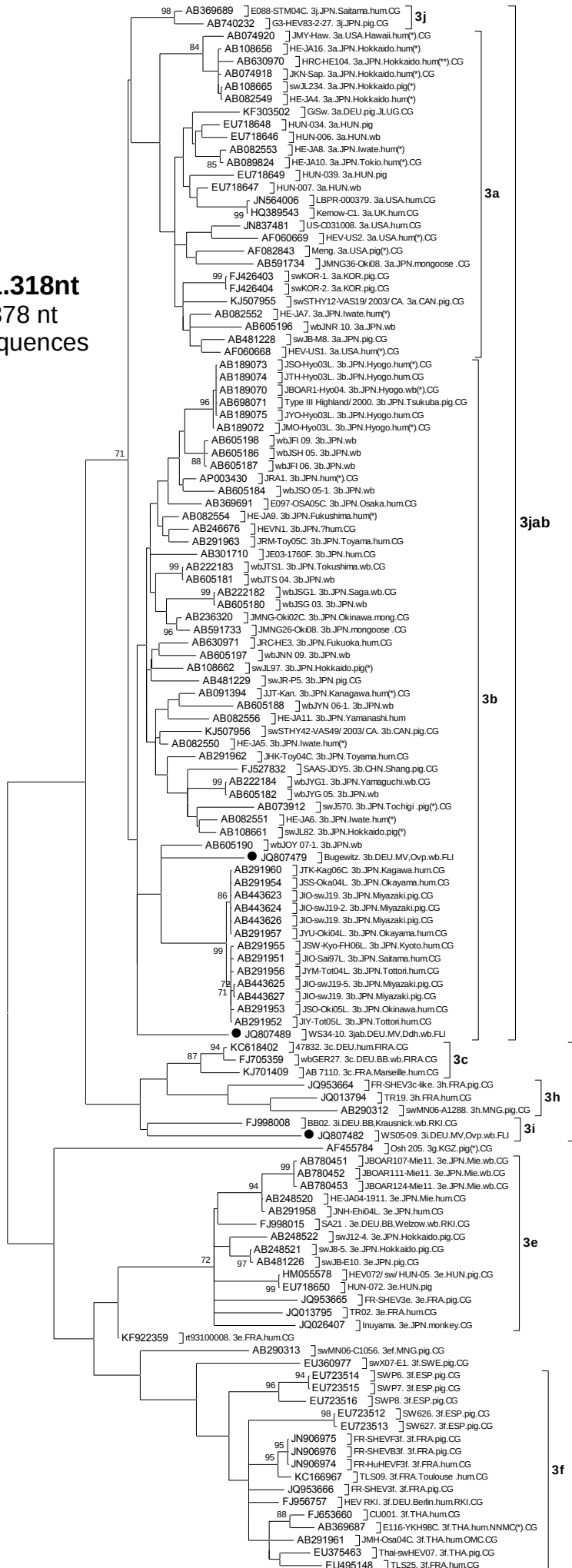

0.05

Supplement: Supplementary file 1 [file viruses-07-02704-s001.zip › viruses-84017-supplementary/Supplem.Fig S02. for Fig3c-right.ORF1.318nt (Burma.M73218 - 61-378 nt) details.pdf]

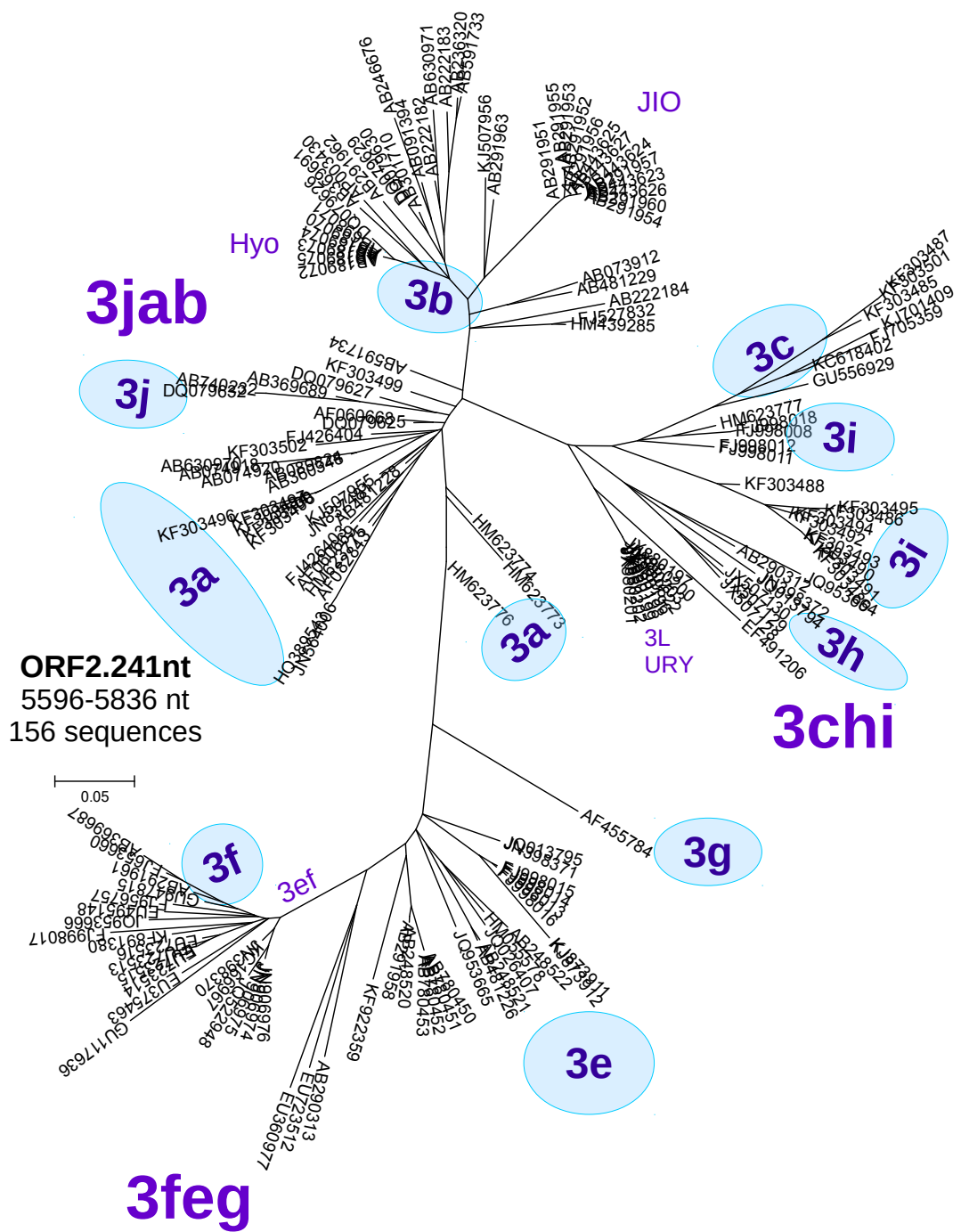

Supplem Figure 03c. ORF2.241nt: 156 partial HEV-3 genome sequences spanning the region Burma.M73218 : 5596-5836 nt

Supplement: Supplementary file 1 [file viruses-07-02704-s001.zip › viruses-84017-supplementary/Supplem.Fig S03abc.pdf]

665 sequence



0.1

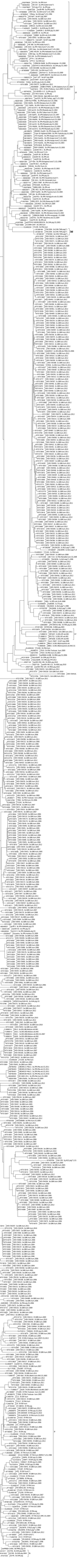

Supplement: Supplementary file 1 [file viruses-07-02704-s001.zip › viruses-84017-supplementary/Supplem.Fig S05. for FigS03b-left.ORF2.280nt (Burma.M73218 - 6017-6296 nt) details.pdf]

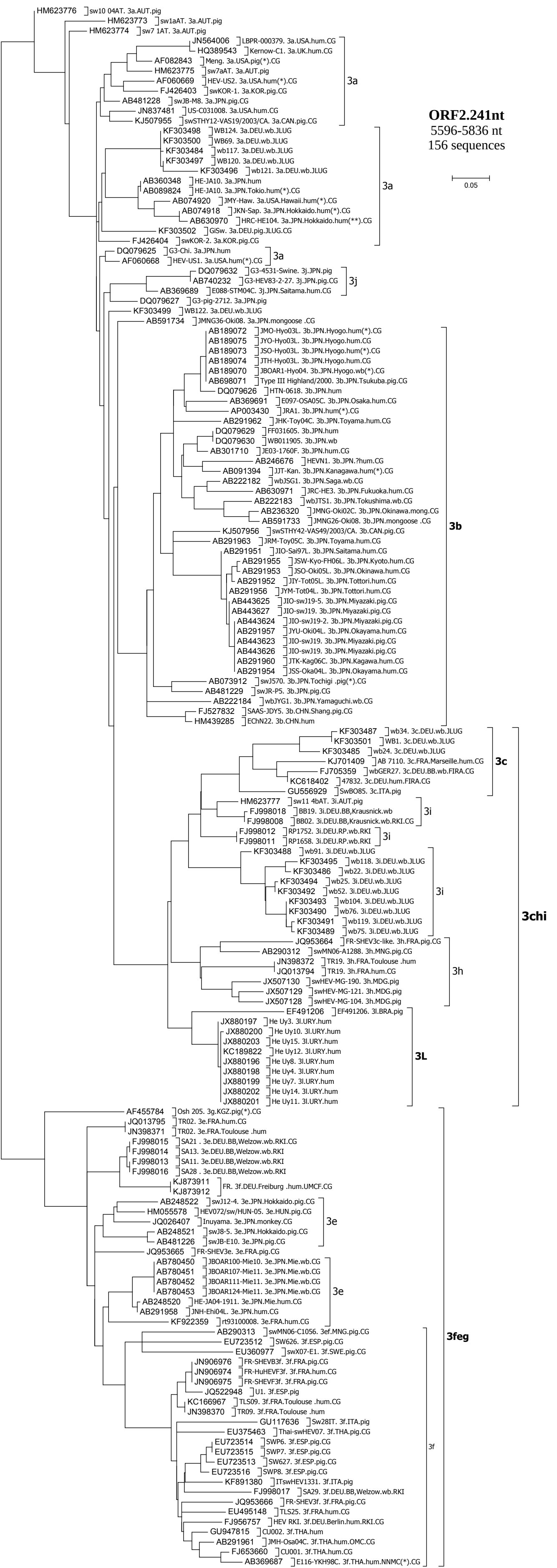

Supplement: Supplementary file 1 [file viruses-07-02704-s001.zip › viruses-84017-supplementary/Supplem.Fig S06. for FigS03c.ORF2.241n (Burma.M73218 - 5596-5836 nt) details.pdf]

HVR.319nt  
2094-2412 nt  
126 sequences

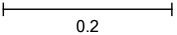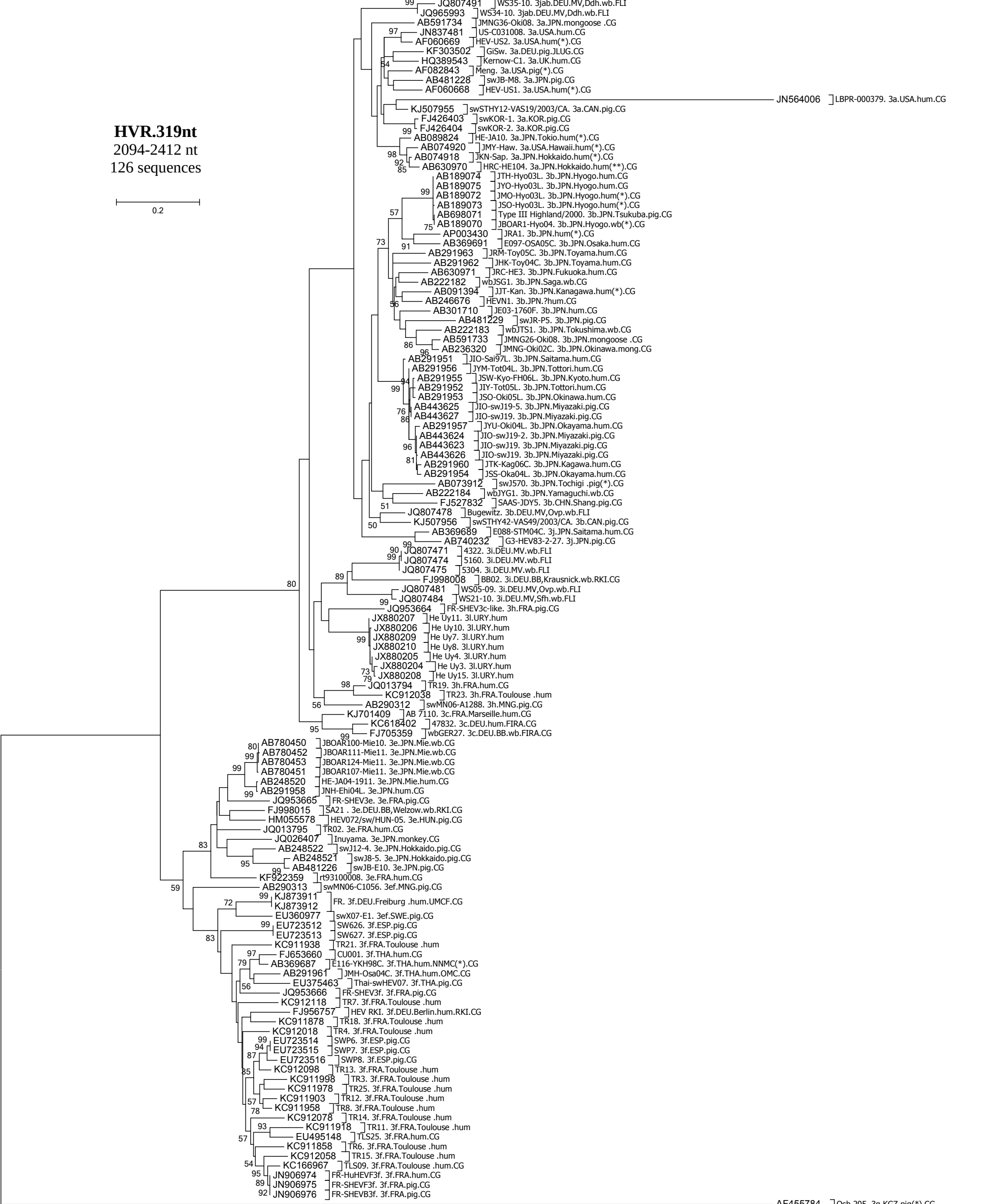

Supplement: Supplementary file 1 [file viruses-07-02704-s001.zip › viruses-84017-supplementary/Supplem.Fig S07. for Fig4a-left.HVR.319nt (Burma.M73218 - 2094-2412 nt) details.pdf]

**RdRp.280nt**  
4284-4563 nt  
147 sequences

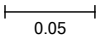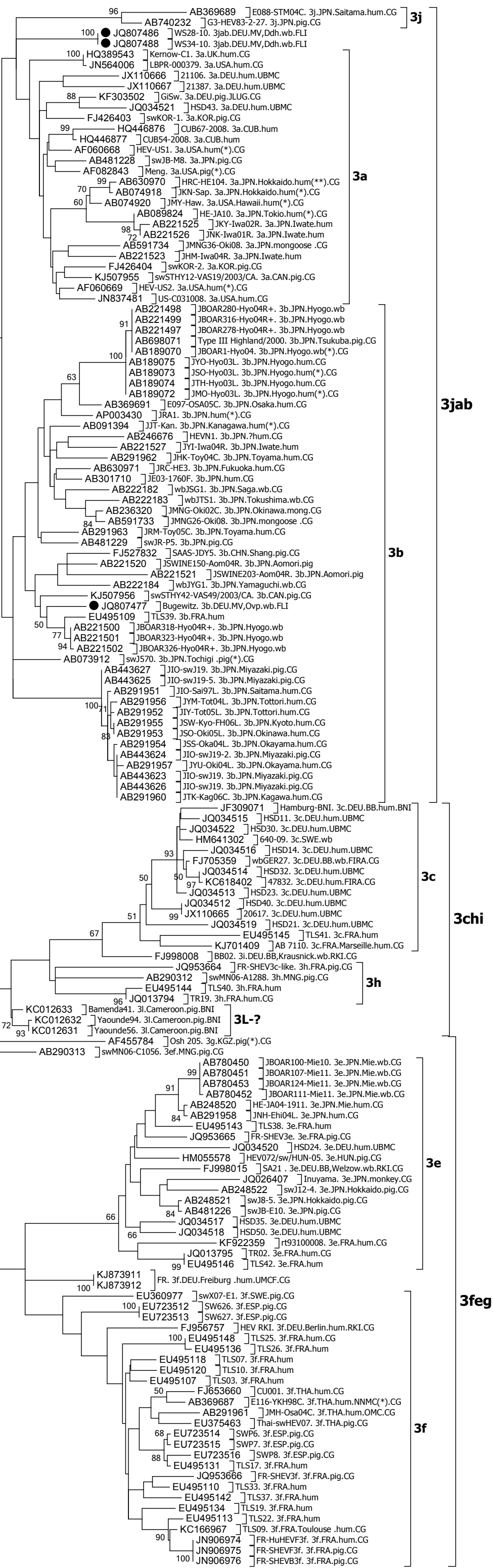

Supplement: Supplementary file 1 [file viruses-07-02704-s001.zip › viruses-84017-supplementary/Supplem.Fig S08. for Fig4b-center.RdRp.280nt (Burma.M73218 - 4284-4563 nt) details.pdf]

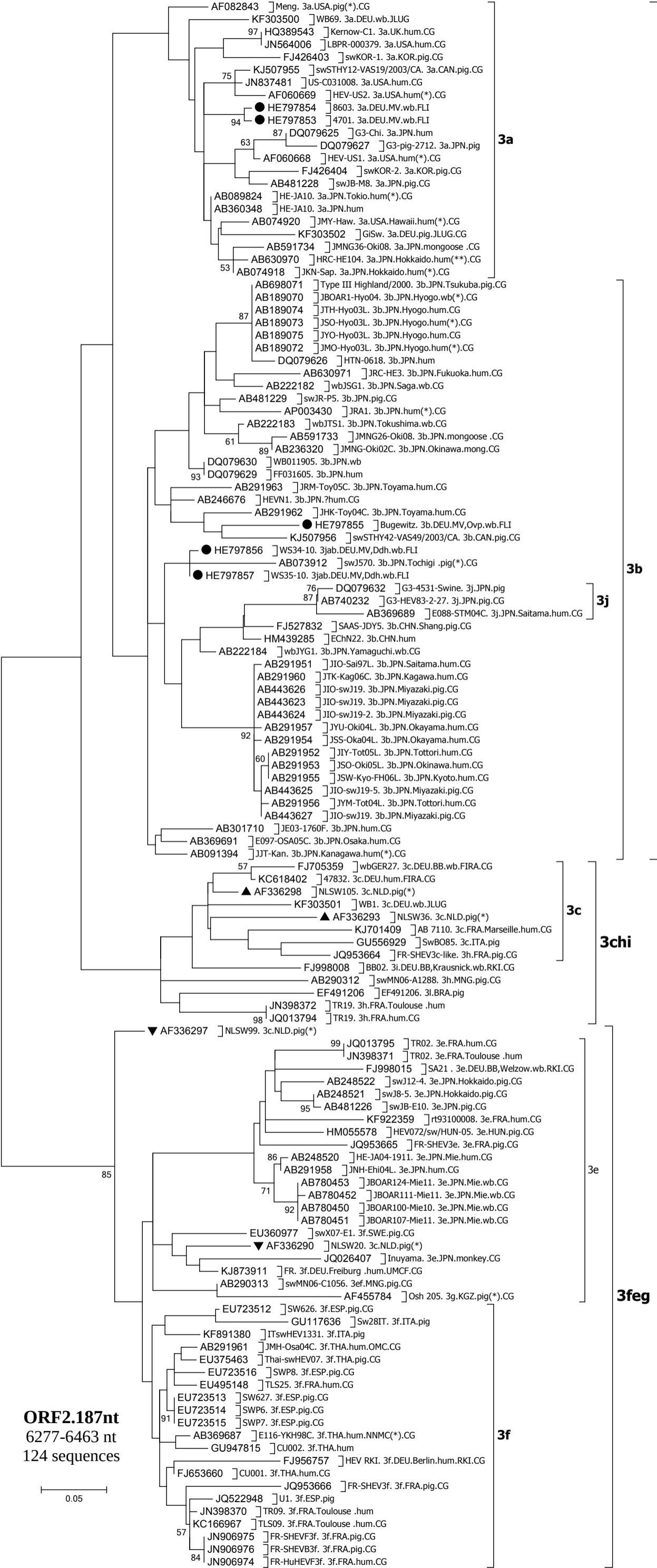

ORF2.187nt  
6277-6463 nt  
124 sequences

0.05

Supplement: Supplementary file 1 [file viruses-07-02704-s001.zip › viruses-84017-supplementary/Supplem.Fig S09. for Fig4c-right.HEV.ORF2.187nt (Burma.M73218 - 6277-6463 nt) details.pdf]

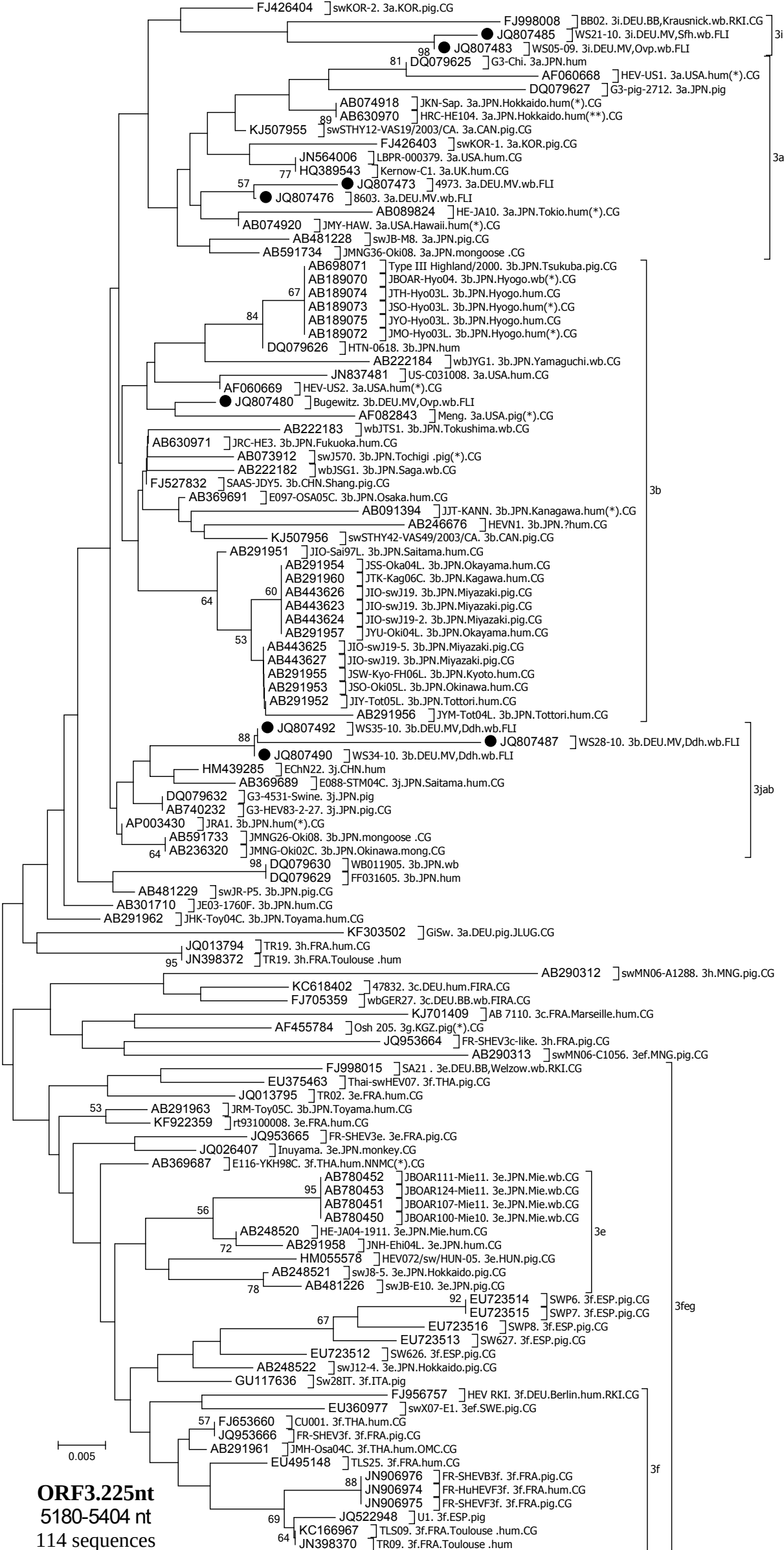

**ORF3.225nt**  
**5180-5404 nt**  
**114 sequences**

Supplement: Supplementary file 1 [file viruses-07-02704-s001.zip › viruses-84017-supplementary/Supplem.Fig S10.ORF3.255nt.NJx1000.only 114 HEV-3.details.pdf]

A horizontal line with vertical end caps, labeled "0.005" below it.

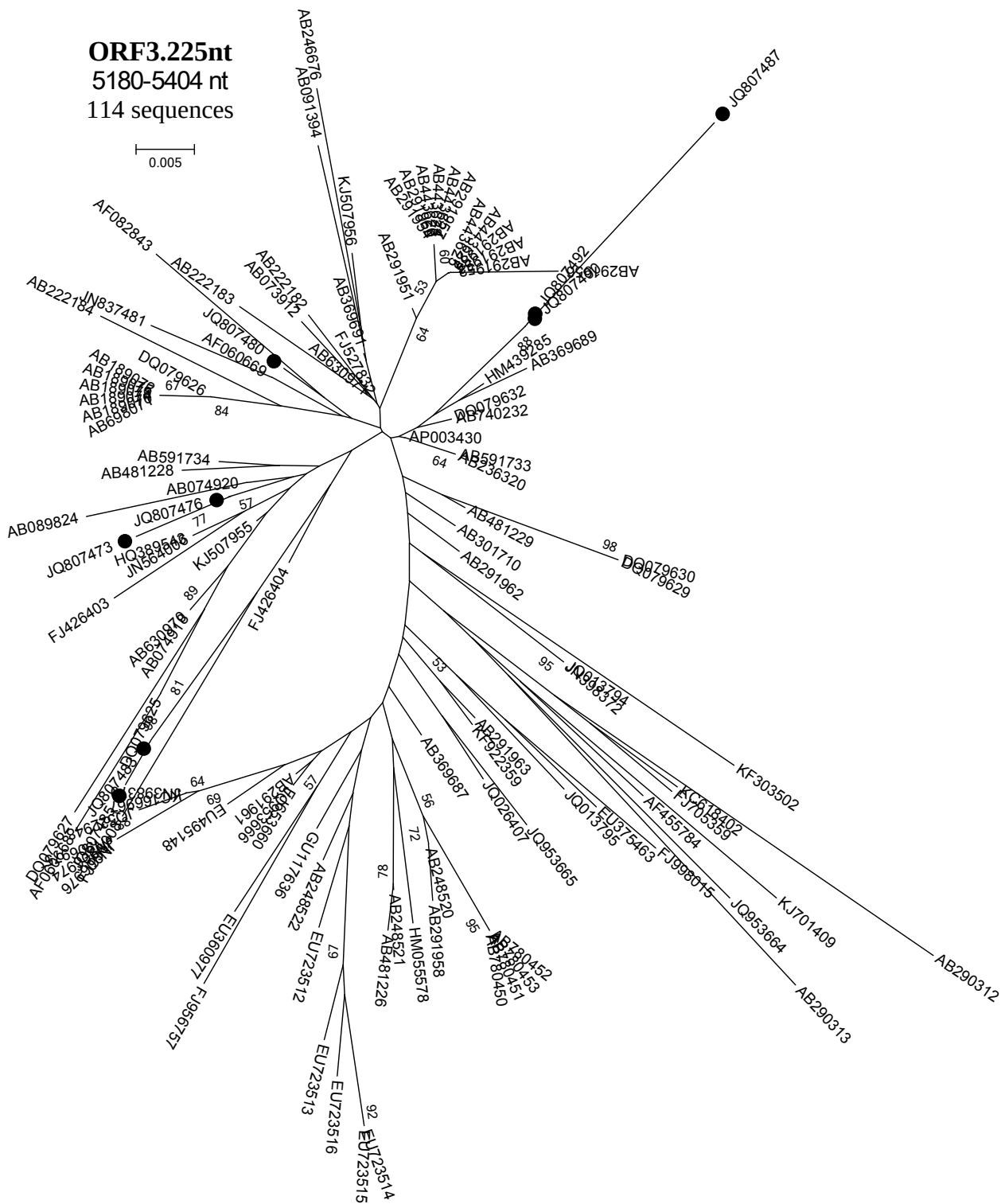

Supplement: Supplementary file 1 [file viruses-07-02704-s001.zip › viruses-84017-supplementary/Supplem.Fig S11.ORF3.255nt.NJx1000.only 114 HEV-3.rad.pdf]

**OFR1.371nt**  
80-450 nt

0.02

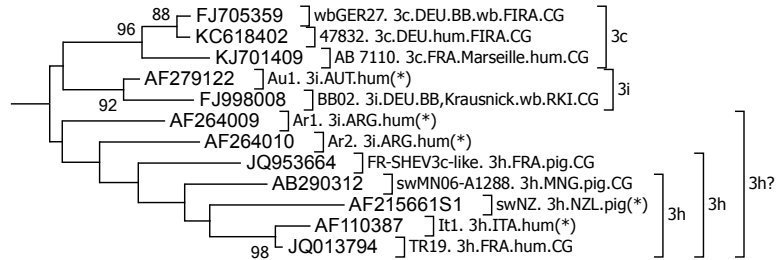

**OFR2.768nt**  
5504-6271 nt

0.02

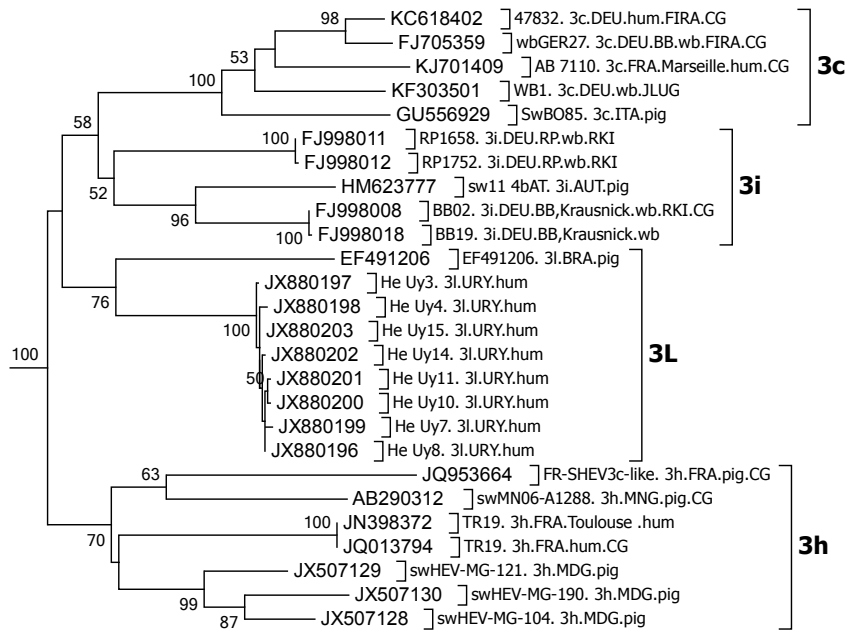

**OFR1.242nt**  
125-366 nt

0.02

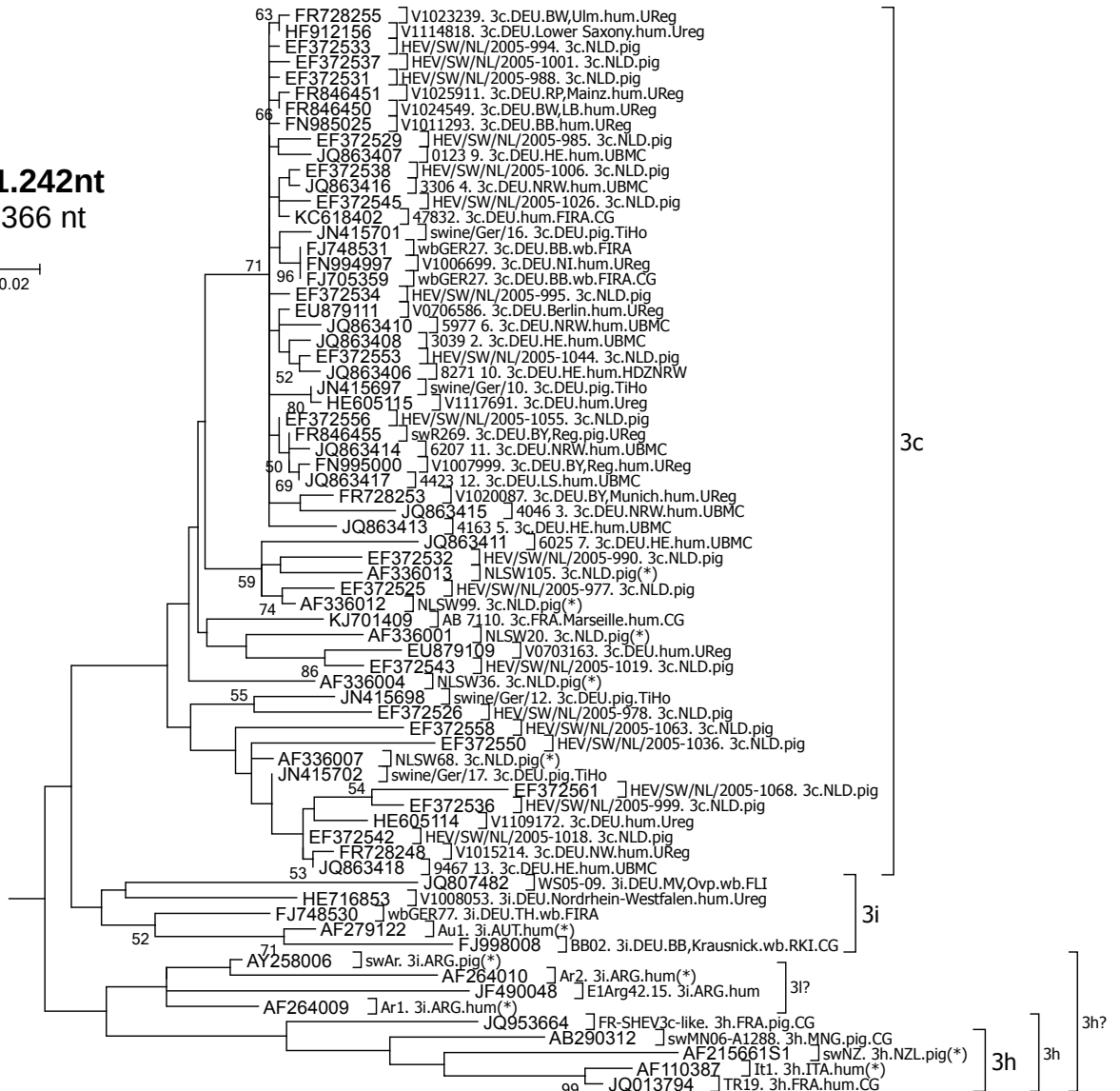

Supplement: Supplementary file 1 [file viruses-07-02704-s001.zip › viruses-84017-supplementary/Supplem.Fig S12. Supplementary figure 3chi details.pdf]

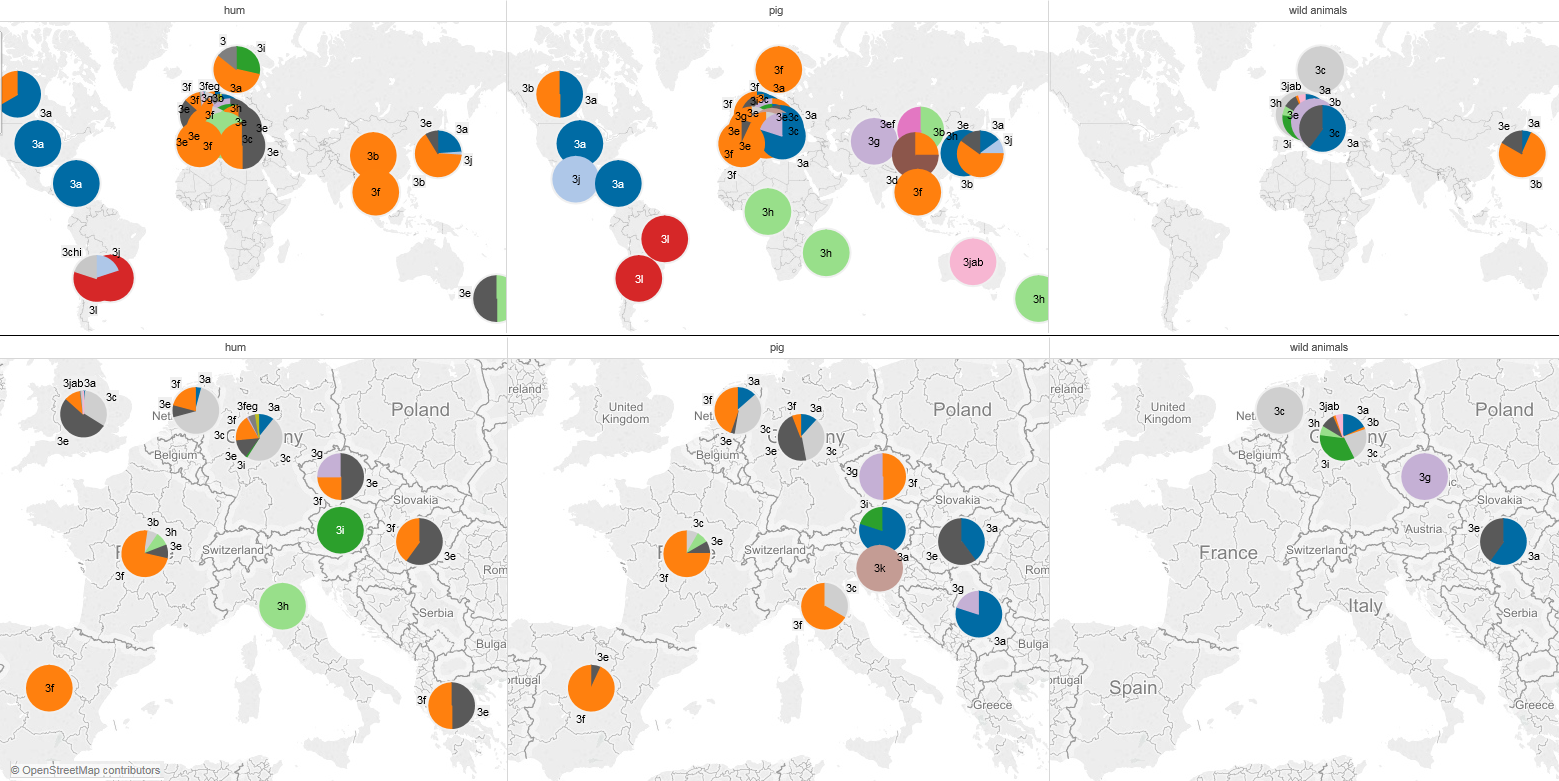

Supplement: Supplementary file 1 [file viruses-07-02704-s001.zip › viruses-84017-supplementary/Supplem.Fig S13. Geographical distribution of subtypes by host (human, domestic pig, or wild animals).png]
