# Supplementary material for: Hepatitis E Virus Genotype 3 Diversity: Phylogenetic Analysis and Presence of Subtype 3b in Wild Boar in Europe
Source: Viruses. 2015 May 22;7(5):2704–26. doi: 10.3390/v7052704 (PMC4452927; doi:10.3390/v7052704)
Supplement: Supplementary file 1 [file Supplem.Fig S04. for FigS03a.ORF1.242nt (Burma.M73218 - 125-366 nt) details.pdf › viruses-84017-supplementary/Supplem.Fig S04. for FigS03a.ORF1.242nt (Burma.M73218 - 125-366 nt) details.pdf]

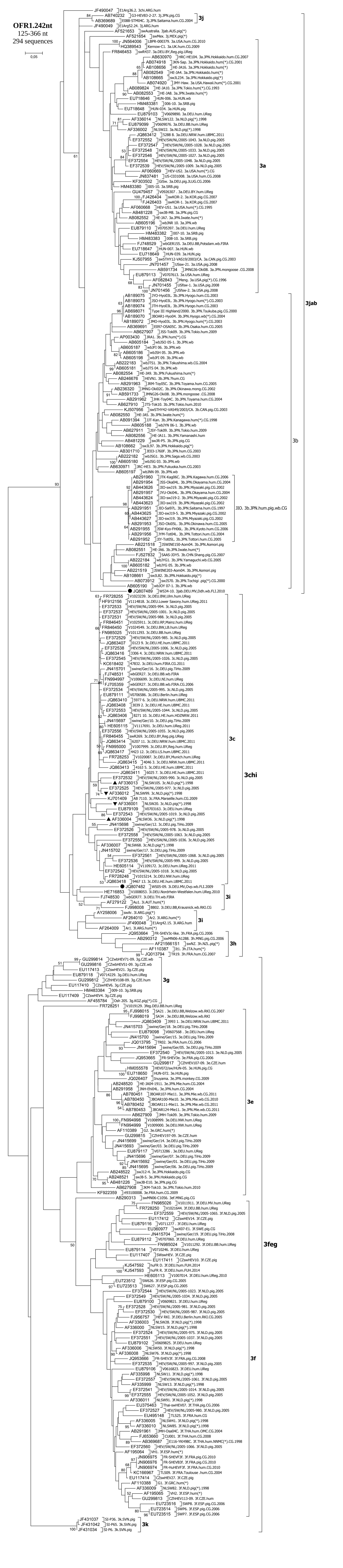
OFR1.242nt
125-366 nt
294 sequences
0.05
3j
3a
3jab
3b
3c
3chi
3i
3h
3g
3e
3feg
3f
3k
